# Nonprobability Web Surveys to Measure Sexual Behaviors and Attitudes in the General Population: A Comparison With a Probability Sample Interview Survey

**DOI:** 10.2196/jmir.3382

**Published:** 2014-12-08

**Authors:** Bob Erens, Sarah Burkill, Mick P Couper, Frederick Conrad, Soazig Clifton, Clare Tanton, Andrew Phelps, Jessica Datta, Catherine H Mercer, Pam Sonnenberg, Philip Prah, Kirstin R Mitchell, Kaye Wellings, Anne M Johnson, Andrew J Copas

**Affiliations:** ^1^London School of Hygiene & Tropical MedicineLondonUnited Kingdom; ^2^University College LondonLondonUnited Kingdom; ^3^University of MichiganAnn Arbor, MIUnited States; ^4^NatCen Social ResearchLondonUnited Kingdom

**Keywords:** Internet survey, Web survey, survey methods, sampling bias, selection bias, sexual behavior

## Abstract

**Background:**

Nonprobability Web surveys using volunteer panels can provide a relatively cheap and quick alternative to traditional health and epidemiological surveys. However, concerns have been raised about their representativeness.

**Objective:**

The aim was to compare results from different Web panels with a population-based probability sample survey (n=8969 aged 18-44 years) that used computer-assisted self-interview (CASI) for sensitive behaviors, the third British National Survey of Sexual Attitudes and Lifestyles (Natsal-3).

**Methods:**

Natsal-3 questions were included on 4 nonprobability Web panel surveys (n=2000 to 2099), 2 using basic quotas based on age and sex, and 2 using modified quotas based on additional variables related to key estimates. Results for sociodemographic characteristics were compared with external benchmarks and for sexual behaviors and opinions with Natsal-3. Odds ratios (ORs) were used to express differences between the benchmark data and each survey for each variable of interest. A summary measure of survey performance was the average absolute OR across variables. Another summary measure was the number of key estimates for which the survey differed significantly (at the 5% level) from the benchmarks.

**Results:**

For sociodemographic variables, the Web surveys were less representative of the general population than Natsal-3. For example, for men, the average absolute OR for Natsal-3 was 1.14, whereas for the Web surveys the average absolute ORs ranged from 1.86 to 2.30. For all Web surveys, approximately two-thirds of the key estimates of sexual behaviors were different from Natsal-3 and the average absolute ORs ranged from 1.32 to 1.98. Differences were appreciable even for questions asked by CASI in Natsal-3. No single Web survey performed consistently better than any other did. Modified quotas slightly improved results for men, but not for women.

**Conclusions:**

Consistent with studies from other countries on less sensitive topics, volunteer Web panels provided appreciably biased estimates. The differences seen with Natsal-3 CASI questions, where mode effects may be similar, suggest a selection bias in the Web surveys. The use of more complex quotas may lead to some improvement, but many estimates are still likely to differ. Volunteer Web panels are not recommended if accurate prevalence estimates for the general population are a key objective.

## Introduction

 Over the past decade, there has been dramatic growth in both Europe and the United States in the use of Web surveys for market research and opinion polling. However, the Web has not been widely used for collecting epidemiological or health surveillance data (or for academic research more generally) despite increasing interest in doing so [1-12]. This is largely due to the reliance on volunteer Web panels (at least when examining the general population) and well-founded concerns about the representativeness of such nonprobability Web surveys that rely on these panels [13].

Volunteer Web panels typically include hundreds of thousands of potential participants who have signed up to participate in Web surveys, often for a small incentive (eg, a payment or points that can be redeemed for goods). Although there are a few Web panels in the United States (the GfK Knowledge Panel) and in Europe (eg, the Longitudinal Internet Study for the Social sciences [LISS] panel in the Netherlands and the German Internet Panel) selected by using probability-based sampling methods, most Web panels are made up of self-selected volunteers who are recruited using a variety of methods (eg, through email databases, visitors to websites, online advertisements). Aside from sampling issues, the main concerns about Web panels include coverage bias because individuals or households without access to the Internet are excluded, and nonresponse bias because response rates to Web surveys are often very low if reported at all [13-16].

Despite these concerns, the use of Web surveys is likely to continue to increase in Britain and elsewhere because they purportedly allow efficient, relatively cheap, and quick data collection [14,17-20], advantages which will increasingly appeal to academic and government researchers faced with rising costs for traditional face-to-face and telephone interview surveys at a time of constrained budgets (eg, see the UK government’s rationale for moving the Community Life Survey to Web data collection [21]) . Web surveys may even be advantageous for certain types of studies, such as those concerned with sensitive behaviors, because interviewers are not present and the greater privacy provided by Web surveys may lead to higher reporting of socially undesirable behaviors [3,8,10,12,19,22,23]. Therefore, it is important to continue to evaluate the representativeness of volunteer Web panels and the circumstances in which they can be used, and to attempt to develop approaches and estimation methods to improve the robustness of Web panel data.

This paper makes a number of contributions to the evidence base by comparing results from 4 volunteer Web panel surveys with those from the third British National Survey of Sexual Attitudes and Lifestyles (Natsal-3), a probability sample interview survey. Firstly, unlike much of the previous research on this topic, which generally compares results for voting intentions or opinion questions, Natsal-3 includes primarily behavioral measures along with a few opinion questions. Secondly, Natsal-3 includes a lengthy computer-assisted self-interview (CASI) component that, like a Web survey, requires participants to enter their answers directly onto a computer. Hence, this study makes a unique contribution by comparing results for sensitive behaviors between Web surveys and a CASI probability sample survey. Thirdly, the study included an experiment that modified the quota controls for 2 of the Web surveys to see whether this would improve the representativeness of their results. Fourthly, although there has been considerable research in the United States and Europe comparing results from Web panel surveys with those from traditional face-to-face, telephone, and mail surveys (much of which is summarized in the 2010 report from the American Association for Public Opinion Research [AAPOR] [13]), there has been very little published research on this topic for British Web panels, despite all the major British market research agencies maintaining large databases of Web panel members.

## Methods

### Data Collection

The 4 Web surveys were carried out by 3 well-known survey organizations in the United Kingdom, each involved in social and market research, and each with large volunteer Web panels. Results from the Web surveys were compared with Natsal-3 results and, for a limited number of variables, with external data. Our focus was not on the individual panels and confidentiality agreements with the companies prohibit us from identifying them.

Natsal is one of the largest surveys on sexual behavior in the world, having interviewed 18,876 adults in 1990-1991 (Natsal-1), 12,110 adults in 1999-2001 (Natsal-2), and 15,162 adults in 2010-2012 (Natsal-3). Natsal-3 involved a stratified, clustered probability sample design and an interview with 1 randomly selected adult aged 16-74 years by a trained interviewer in the participant’s own home. Median interview length was 53 minutes, split (approximately equally) between a face-to-face computer-assisted personal interview (CAPI) and CASI for the most sensitive questions. Multimedia Appendix 1 shows which questionnaire sections in Natsal-3 were asked in CAPI and which were asked in CASI. Fieldwork was carried out between September 2010 and August 2012, and achieved a response rate of 57.7% (similar to Natsal-2 for the comparable age group) [24].

A subset of approximately 130 Natsal-3 questions was included on the Web surveys, which took approximately 20 minutes on average to complete. The Web survey questions were exactly the same as those asked in Natsal-3, except where changes in format were required (eg, where show cards were used in Natsal-3). A version of the Web survey questionnaire is included in Multimedia Appendix 2. The age range included in the Web surveys was restricted to adults aged 18-44 years (n=8969) and all analyses in this paper are restricted to that age range.

Two of the Web surveys set “basic” quotas on variables which are central to measuring sexual behavior (age and partnership status), whereas the other 2 involved “modified” quotas with additional variables not typically used in setting quota controls (eg, opinion variables). Given that the purpose of the study was to see if the modified quotas would bring the Web survey estimates closer to Natsal-3, the modified quotas were set using distributions from Natsal-3 for the relevant variables.

Web survey basic quota 1 (WS-B1, carried out by Company A) and Web survey basic quota 2 (WS-B2, carried out by Company B) aimed to achieve samples of approximately 2000 cases with basic quotas set for age group (18-24, 25-34, and 35-44 years) within sex, partnership status (married/living as married vs all others) within sex, and region (London vs rest of Britain). The quotas for age within sex and region were set by reference to Office for National Statistics (ONS) midyear 2010 population estimates, whereas the quota for partnership status within sex used data from the 2009 British Labour Force Survey [25]. The quotas are shown in Table 1.

**Table 1:** Description of the surveys included in the study.

Sample type and survey		Quotas	Achieved sample size, N (18-44 years)	Variables used for weighting^a^
**Basic quota**				
	WS-B1 (Company A)	Age within sex	2099	Age within sex
		Partnership status within sex		
		Region		
	WS-B2 (Company B)	Age within sex	2000	Age within sex
		Partnership status within sex		
		Region		
**Modified quota**				
	WS-M1 (Company B)	Age within sex	2000	Age within sex
		Partnership status within sex		
		Region		
		Age left full-time education		
		Any under 18s in household		
	WS-M2 (Company C)	Age within sex	2021	Age within sex
		Partnership status within sex		
		Frequency of drinking alcohol		
		Age left full-time education		
		Attitude to same-gender sex		
		Household size		
**Probability**				
	Natsal-3	Not applicable	8969	Age within sex
				Region

^a^ From 2011 Census.

Web survey modified quota 1 (WS-M1), also carried out by Company B, used modified quota controls. These were determined by identifying which sociodemographic characteristics available in Natsal-3 and that Company B also had available for their Web panel members were significantly associated with key behavioral measures (including those in Multimedia Appendix 3). Then, we examined how WS-B2 and Natsal-3 differed in terms of these identified characteristics. For this second process, the WS-B2 dataset was combined with the Natsal-3 dataset (because Natsal-3 fieldwork was not yet complete, this analysis used only the first full year of Natsal-3 fieldwork and included 4459 participants aged 18-44 years). An indicator of whether the participant belonged to Natsal-3 or WS-B2 was then used as the dependent variable in a forward stepwise logistic regression model selection process using *P*<.05 in STATA 12.0 (StataCorp LP, College Station, TX, USA). The variables included in the process were age finished full-time education, participant’s current economic activity, whether there were any residents younger than 18 years living in the participant’s household, household size, and the area-based Index of Multiple Deprivation (IMD) score for the participant’s postcode [26]. The first 3 of these listed variables were selected, but because the first 2 are strongly correlated, age finished full-time education was chosen (as the more statistically significant) along with any residents younger than 18 years in the household as factors to modify the basic quotas for WS-M1; quotas were set so that the distribution for these 2 variables would match those obtained in Natsal-3.

Because market research agencies tend to collect very limited information on all their Web panel members, for Web survey modified quota 2 (WS-M2; carried out by Company C) the aim was to identify a number of variables associated with key measures that are *not* normally available for panel members, but which could be obtained for a large subsample of the panel by including questions on an initial Web omnibus (ie, regular multipurpose) survey. Using Natsal-3 data, significant associations in bivariate analysis between key behaviors (including those in Multimedia Appendix 3) and sociodemographic and attitudinal variables were examined to generate a shortlist of possible questions to be included on Company C’s regular Web omnibus survey. The sociodemographic and attitudinal variables with the highest number of significant associations with key behaviors and which remained significant after adjusting for age, partnership status, and region in the logistic regression analysis were identified as potential variables to be used as additional quotas. WS-M2 then proceeded in 2 stages. First, 6 additional questions (on current smoking status, frequency of drinking alcohol, age completed full-time education, tenure, and attitude toward same-gender sex and abortion) were included on Company C’s Web omnibus. Although the original target was to collect data for these 6 variables for approximately 30,000 members of Company C’s Web panel, data were collected for only 9176 panel members within the 18-44 age range. The second stage used a forward stepwise model with Natsal-3 or the omnibus survey as a binary outcome to indicate which of the 6 variables, along with some basic sociodemographic variables held by the agency, best modeled the difference between the 2 surveys. Four variables came out as highly significant (frequency of drinking alcohol, age completed education, attitude toward same-gender sex, and household size) and were selected as additional factors to form quotas (along with age and partnership status within sex) for WS-M2. Quotas were set so that the distributions of these 4 variables would match those obtained in Natsal-3. Because it was not possible to fill all the quotas using only the 9176 panel members for whom this additional information was now available, more panel members were invited to participate in WS-M2. Those who had not completed the Web omnibus were filtered into the relevant quotas by answering the additional questions before starting the main questionnaire (and were excluded from the main questionnaire if their quota was already filled).

By the time the full Natsal-3 dataset became available for analysis (in early 2013), data from the 2011 UK population census were also available. Natsal-3 and all Web surveys were poststratified to 2011 census figures for age within sex (which differed slightly from the 2010 midyear population estimates). Natsal-3 data were also weighted by region, but we did not weight the Web surveys by region because the agencies did not collect regional data on the same basis. In the event, weighting by region did not greatly affect the estimates.

Participation in Natsal-3 and the Web surveys was based on fully informed consent. The study was approved by the Oxfordshire Research Ethics Committee A (reference number 12/SC/0070).

### Analysis Methods

Bias regarding participant sociodemographic characteristics was assessed by comparing the estimates from the 4 Web surveys and Natsal-3 with external benchmark data from the 2011 UK population census; the ONS Integrated Household Survey (IHS) for 2011 (except for sexual identity which comes from IHS April 2011-March 2012), a large annual survey of approximately 400,000 individuals used to produce official statistics [27]; or from the 2010 National Travel Survey for benchmarks on holding a driving license [28]. Comparisons were made for a number of key behavioral measures reported in the initial series of Natsal-3 papers published in *The Lancet* [29-32] and also key attitudinal measures. For these measures, Natsal-3 was treated as the benchmark because its results have been widely used within government and academia, and because it is the only probability-based survey measuring these topics in the British population. No independent benchmarks exist for the behavioral and attitudinal variables.

The difference between the benchmark data and each survey for each outcome variable of interest is expressed as the odds ratio (OR). These ORs were obtained from binary or ordinal logistic regression depending on the nature of the outcome (no outcome was continuous). Nonordered categorical variables were reduced to binary form to avoid multiple ORs for 1 variable. The ORs are presented with 95% confidence intervals (CIs). One summary measure of the performance of a survey was the number of key estimates for which the survey differed significantly from the benchmark at the 5% level. Another measure of overall bias for a survey was the average absolute OR across outcomes, where the absolute OR for an OR less than 1 was calculated as 1/OR (eg, an OR of 0.5 is treated as 2.0). Average absolute ORs were also calculated separately for CAPI and CASI and for behavior and opinion questions because the performance of the Web surveys could differ by these question types. Absolute OR was selected in preference to absolute difference because we felt it better reflected the importance of differences across the range of prevalence from rare to common.

Generalized estimating equations were used to test whether the 2 basic quota Web surveys were consistent (ie, gave the same responses to each question). Robust standard errors to reflect the within-person clustering of outcome responses were used. To assess whether either approach to the use of modified quotas resulted in more accurate estimates, the average absolute OR across the outcomes for the 2 basic Web surveys (WS-B1 and WS-B2) was compared with the corresponding average OR for each modified Web survey (WS-M1 and WS-M2). The bootstrap method [33], resampling participants in each Web survey 100 times, was used to obtain a standard error for the average absolute OR for each modified survey and for the 2 basic surveys combined. This focused on the behavior and opinion outcomes because these were of central interest. The uncertainty in Natsal-3 estimates was not considered because it was not relevant to the comparison of Web surveys. The average absolute OR was bounded by 1 and unlikely to be normally distributed, so CIs are not provided but approximate tests for difference between average absolute ORs were performed under the assumption of normality.

## Results

### Meeting the Web Survey Quotas

The age-sex quotas were generally met by all 4 Web surveys, except for young men aged 18-24 years which was undertarget in 2 of the surveys; in WS-B1, 211 of the target number of 267 (79.0%) was achieved and in WS-M2 it was 228 out of 267 (85.4%).

Even with Web panels containing hundreds of thousands of members, both Companies B and C had difficulties meeting the modified quotas. Both WS-M1 and WS-M2 could not find enough people who finished their education before age 17 years. WS-M2 also fell short of the quotas for large (≥4 person) households, infrequent drinkers of alcohol (those who drink less than once a week), and 1 attitude question (tolerance of same-sex relationships). These quotas had to be relaxed to achieve the target of 2000 completed questionnaires (Multimedia Appendix 4).

### Comparing Participant Characteristics With External Benchmarks

**Figure 1:**
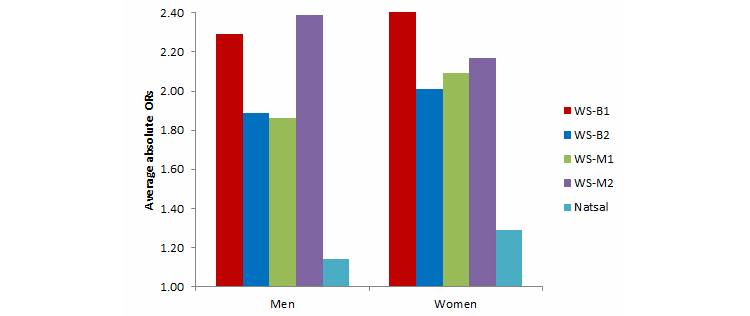
Average absolute odds ratios for participant characteristics for men and women: Web survey basic quota 1 and 2 (WS-B1 and WS-B2), Web survey modified quota 1 and 2 (WS-M1 and WS-M2), and Natsal-3 compared with independent benchmarks.

Estimates regarding participant characteristics from all 4 Web surveys and Natsal-3 were compared with external benchmarks to assess bias. Variables used as quotas for any of the Web surveys were not included, leaving 6 variables to be compared: housing tenure, current economic activity, ethnicity, self-assessed general health, whether the participant had a driving licence valid in the United Kingdom, and sexual identity. The results are summarized in Figure 1 and Table 2; detailed distributions are shown in Multimedia Appendix 5 and the ORs for each variable are shown in Multimedia Appendix 6.

As Figure 1 shows, for both sexes the average absolute OR was much closer to 1 for Natsal-3 than for the Web surveys suggesting that the probability Natsal-3 sample was more representative of the general population. Among the Web surveys, for both sexes, WS-B2 and WS-M1 had the lowest average absolute ORs.

For men, the largest absolute OR was much lower for Natsal-3 (at 1.32) than for the Web surveys (range 3.13-5.23) (Table 2). The pattern was the same for women, although the differences were not as great. The largest absolute OR was for sexual identity across all 4 Web surveys and for women in Natsal-3 (for men in Natsal-3 it was general health).

The evidence that Web surveys with modified quotas performed better than those with basic quotas was mixed. Among the Web surveys, for men WS-M1 had the lowest average absolute OR, but it was only a small improvement on WS-B2, and both WS-B1 and WS-B2 performed better than the other survey with modified quotas (WS-M2). For women, there was less difference in average absolute ORs between the 4 Web surveys, and the basic quota WS-B2 had the lowest average absolute OR.

**Table 2:** Summary of participant characteristics for men and women: Web survey basic quota 1 and 2 (WS-B1 and WS-B2), Web survey modified quota 1 and 2 (WS-M1 and WS-M2), and Natsal-3 compared with independent benchmarks.^a^

Summary of patient characteristics compared with benchmarks		WS-B1	WS-B2	WS-M1	WS-M2	Natsal-3
**Men**						
	Average absolute OR (from benchmarks)	2.29	1.89	1.86	2.39	1.14
	Largest absolute OR	5.23	3.45	3.13	3.66	1.32
	N significantly different from benchmarks	3/6	5/6	4/6	5/6	2/6
**Women**						
	Average absolute OR (from benchmarks)	2.42	2.01	2.09	2.17	1.29
	Largest absolute OR	4.78	3.94	3.83	3.77	1.91
	N significantly different from benchmarks	4/6	5/6	6/6	5/6	4/6

^a^The sources of the independent benchmarks were Census 2011 for ethnicity, current economic activity, and self-assessed general health; IHS 2011 for housing tenure and sexual identity; and National Travel Survey 2010 for current driving licence valid in the United Kingdom.

### Comparing Key Estimates from the Web Surveys With Natsal-3 Benchmarks

**Figure 2:**
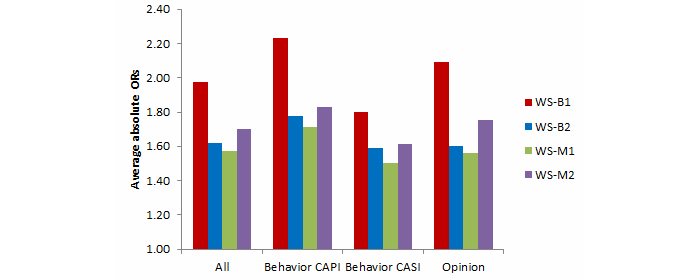
Men: Summary of average absolute ORs for behavior (CAPI and CASI) and opinion variables for the 4 Web surveys compared with Natsal-3.

**Figure 3:**
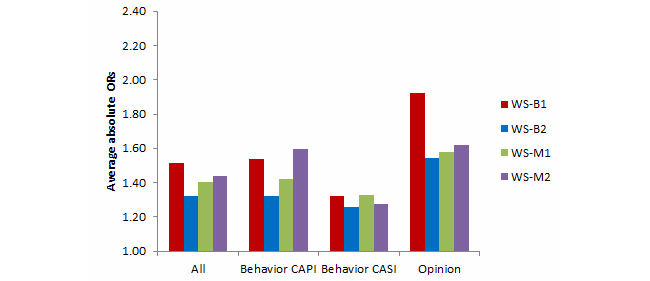
Women: Summary of average absolute ORs for behavior (CAPI and CASI) and opinion variables for the 4 Web surveys compared with Natsal-3.

Comparisons between the Web surveys and Natsal-3 benchmarks for key estimates are summarized in Table 3 by mode (ie, whether the Natsal-3 question was asked in CAPI or CASI) and question type (behavior or opinion) and are shown graphically in Figures 2 and 3. All 4 Web surveys were substantially different from the Natsal-3 benchmarks, as shown in Multimedia Appendix 3 for behaviors and in Multimedia Appendix 7 for opinions, and the ORs for each variable are shown in Multimedia Appendix 8.

**Figure 4:**
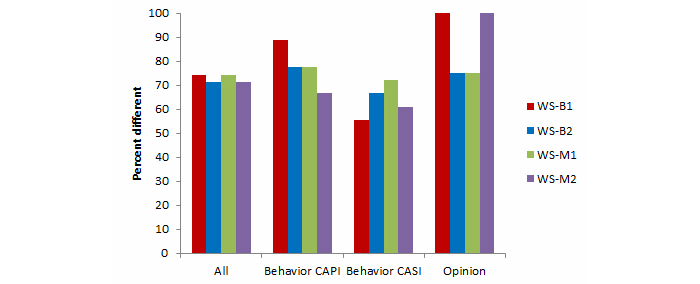
Men: Percentage of variables (all: n=35; behavior CAPI: n=9; behavior CASI: n=18; opinion: n=8) in 4 Web surveys significantly different from Natsal-3.

**Figure 5:**
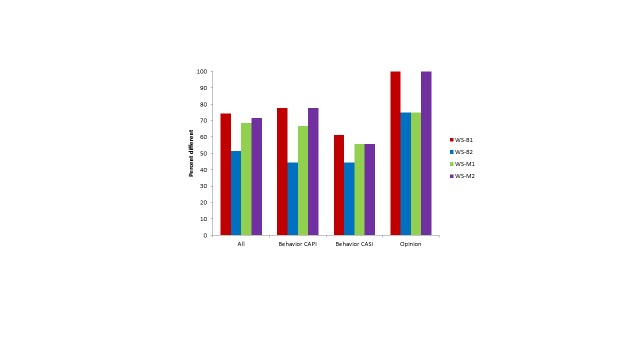
Women: Percentage of variables (all: n=35; behavior CAPI: n=9; behavior CASI: n=18; opinion: n=8) in 4 Web surveys significantly different from Natsal-3.

The average absolute ORs ranged from 1.32 (WS-B2 women) to 1.98 (WS-B1 men). In all Web surveys, the average absolute ORs were lower for women than for men. Several individual estimates showed very large differences, especially for men; for example, the percentage of men who reported having 1 or more same-sex partners in the past 5 years was 3.0% in Natsal-3 compared with a range of 7.9% to 12.9% in the Web surveys (with ORs ranging from 2.81 to 4.85). Moreover, for each Web survey, a majority of the variables examined were significantly different from the Natsal-3 benchmarks (Figures 4 and 5). The highest percentage of differences between the Web surveys and Natsal-3 was found for the opinion questions (primarily 5-point scales). In 2 of the Web surveys, all 8 opinion questions were significantly different from Natsal-3.

As with sociodemographic characteristics, the evidence that Web surveys with modified quotas performed better than those with basic quotas was mixed. Compared with WS-B1, both WS-M1 and WS-M2 had results (for average absolute ORs) closer to Natsal-3 for both sexes. Compared with WS-B2, however, WS-M1 results showed larger differences in average absolute ORs than WS-B2 for women (but not for men). And, for both sexes, WS-B2 had lower average absolute ORs than did WS-M2. The evidence remains mixed when looking at the different question types and survey modes; for example, WS-M1 had the lowest average absolute ORs for the behavior (whether CAPI or CASI) questions for men, but not for women. Overall, there was not one Web survey that consistently performed better than the others across sex, survey mode, or question type.

Performing tests to compare results from the combined basic quota Web surveys with each modified quota Web survey revealed that the only statistically significant improvement overall (reduction in average absolute OR) was for men in WS-M1 (Table 4).

Another important concern is not only how results from Web panel surveys compare with those using other modes of data collection, but also whether the results obtained are likely to vary significantly according to which Web panel is used. When the 2 basic quota Web surveys were compared using generalized estimating equations, the results were significantly different (*P*<.001) and differences were often appreciable, as can be seen in in Multimedia Appendix 3.

**Table 3:** Summary of behavior (CAPI/CASI) and opinion (CAPI/CASI) variables for Web survey basic quota 1 and 2 (WS-B1 and WS-B2) and Web survey modified quota 1 and 2 (WS-M1 and WS-M2) compared with Natsal-3 for men and women.

Summary of behavior and opinion variables compared with Natsal-3		WS-B1	WS-B2	WS-M1	WS-M2
**Men, average absolute OR (SE)** ^a^					
	All	1.98 (0.06)	1.62 (0.04)	1.57 (0.03)	1.70 (0.05)
	Behavior CAPI	2.24 (0.08)	1.78 (0.05)	1.71 (0.06)	1.83 (0.06)
	Behavior CASI	1.80 (0.07)	1.59 (0.05)	1.50 (0.04)	1.61 (0.07)
	Opinion CAPI	2.18 (0.11)	1.47 (0.06)	1.43 (0.06)	1.72 (0.09)
	Opinion CASI	1.95 (0.10)	1.83 (0.09)	1.79 (0.10)	1.81 (0.10)
**Men, largest absolute OR (SE)** ^a^					
	All	4.85 (0.45)	3.28 (0.38)	2.89 (0.28)	3.78 (0.42)
	Behavior CAPI	4.54 (0.35)	3.11 (0.24)	2.87 (0.25)	3.75 (0.28)
	Behavior CASI	4.85 (0.45)	3.28 (0.38)	2.89 (0.28)	3.78 (0.42)
	Opinion CAPI	2.58 (0.15)	2.19 (0.16)	2.28 (0.17)	2.15 (0.16)
	Opinion CASI	2.54 (0.15)	2.18 (0.17)	2.24 (0.18)	2.12 (0.17)
**Men, significantly different from Natsal-3, n/N**					
	All	26/35	25/35	26/35	25/35
	Behavior CAPI	8/9	7/9	7/9	6/9
	Behavior CASI	10/18	12/18	13/18	11/18
	Opinion CAPI	5/5	4/5	3/5	5/5
	Opinion CASI	3/3	2/3	3/3	3/3
**Women, average absolute OR (SE)** ^a^					
	All	1.51 (0.03)	1.32 (0.02)	1.40 (0.03)	1.44 (0.03)
	Behavior CAPI	1.54 (0.04)	1.32 (0.04)	1.42 (0.05)	1.59 (0.05)
	Behavior CASI	1.32 (0.04)	1.26 (0.03)	1.31 (0.03)	1.28 (0.03)
	Opinion CAPI	1.89 (0.13)	1.43 (0.07)	1.42 (0.06)	1.43 (0.06)
	Opinion CASI	1.97 (0.11)	1.72 (0.09)	1.84 (0.11)	1.95 (0.11)
**Women, largest absolute OR (SE)** ^a^					
	All	2.70 (0.14)	2.04 (0.16)	2.09 (0.14)	2.65 (0.16)
	Behavior CAPI	2.70 (0.14)	2.04 (0.16)	2.09 (0.14)	2.65 (0.16)
	Behavior CASI	1.91 (0.16)	1.69 (0.18)	1.88 (0.17)	1.82 (0.16)
	Opinion CAPI	2.44 (0.34)	1.93 (0.13)	1.92 (0.20)	1.85 (0.17)
	Opinion CASI	2.02 (0.17)	1.79 (0.15)	1.91 (0.15)	2.20 (0.16)
**Women, significantly different from Natsal-3, n/N**					
	All	26/35	18/35	24/35	25/35
	Behavior CAPI	7/9	4/9	6/9	7/9
	Behavior CASI	11/18	8/18	10/18	10/18
	Opinion CAPI	5/5	3/5	3/5	5/5
	Opinion CASI	3/3	3/3	3/3	3/3

^a^ Reference Natsal-3.

**Table 4:** Comparison of combined basic quota (WS-B1/WS-B2) Web surveys with first (WS-M1) and second (WS-M2) modified quota Web surveys for behavior and opinion (CAPI/CASI) variables for men and women.

Comparison of basic quota with modified quota Web surveys		WS-B1/WS-B2 combined	WS-M1	*P* ^a^	WS-M2	*P* ^a^
**Men, average absolute OR** ^b^ **(SE)**						
	All	1.80 (0.04)	1.57 (0.03)	<.001	1.70 (0.05)	.10
	Behavior CAPI	2.01 (0.05)	1.71 (0.06)	<.001	1.83 (0.06)	.02
	Behavior CASI	1.70 (0.05)	1.50 (0.04)	<.001	1.61 (0.07)	.26
	Opinion CAPI	1.83 (0.07)	1.43 (0.06)	<.001	1.72 (0.09)	.31
	Opinion CASI	1.89 (0.07)	1.79 (0.10)	.40	1.81 (0.10)	.52
**Women, average absolute OR** ^b^ **(SE)**						
	All	1.42 (0.02)	1.40 (0.03)	.54	1.44 (0.03)	.55
	Behavior CAPI	1.43 (0.03)	1.42 (0.05)	.85	1.59 (0.05)	.01
	Behavior CASI	1.29 (0.02)	1.31 (0.03)	.62	1.28 (0.03)	.79
	Opinion CAPI	1.66 (0.08)	1.42 (0.06)	.01	1.43 (0.06)	.01
	Opinion CASI	1.85 (0.07)	1.84 (0.11)	.94	1.95 (0.11)	.45

^a^ *P* values were calculated using the bootstrap method as described in the Methods section.

^b^ From benchmarks.

## Discussion

As the first study in Britain to compare surveys using volunteer Web panels maintained by different research agencies, we demonstrate that, as in other countries, they are not reliably able to provide scientifically robust results. Between 60% and 75% of key estimates in each of 4 Web surveys were significantly different from the benchmarks provided by Natsal-3, a high-quality probability sample survey. Differences between the Web surveys and Natsal-3 were very large for some key behaviors, such as estimates of same-sex sexual experience, which were 2 to 3 times higher in the Web surveys. There were important differences in sample composition as well; for example, compared with both Natsal-3 and the general population, the Web surveys contained more men and women who self-identified as gay/bisexual and fewer men and women from nonwhite ethnic groups.

One limitation of this study is that it is not possible to determine the extent to which the differences observed between the Web surveys and Natsal-3 were due to differences in sample composition or to differences in mode of data collection. Another limitation is that, for the key estimates of sexual behavior, it is not possible to say definitively whether Natsal-3 or the Web surveys provide the most “accurate” estimates. However, we view Natsal-3 as likely to be less biased given the comparisons with independent benchmarks, which showed that participants in the Web panel surveys were less representative of the general population on a number of sociodemographic and other characteristics than Natsal-3 participants. This was found even for the relatively young population (18 to 44 years) included in this study. This is an age group which does not suffer from undercoverage due to lack of Internet use or access because more than 90% of the 18-44 years age group in Britain use the Internet at least once a week and live in households with Internet access [34]. Differences in sample composition, therefore, are likely to explain at least some of the differences observed in the behavioral estimates between the Web surveys and Natsal-3. Further, our findings suggest that setting quotas on key demographics (eg, age), or even on variables known to be correlated to key estimates, are unlikely to ensure representativeness on demographic (or other) variables that are not used to set quotas.

The average absolute ORs in the Web surveys were higher for the Natsal-3 behavior variables asked in CAPI than in CASI. This is consistent with the possibility that Web surveys, because of the greater anonymity afforded by the Web, may obtain higher rates of disclosure of sensitive behaviors than a CAPI survey, and that fewer differences would be expected between a Web survey and sensitive questions asked in CASI. However, even though both CASI and Web surveys are self-administered, they differ in other respects, such as the presence of an interviewer and the degree of perceived privacy. Although the Web surveys obtained much higher rates of disclosure of same-sex attraction and experience than obtained in either CAPI or CASI in Natsal-3, over a range of variables there was no consistent pattern found in whether reports of sensitive behaviors were higher in the Web surveys or in CASI. For some variables, there were only small differences, or indeed lower reporting, in the Web surveys than in Natsal-3 CASI. We found that approximately one-half to two-thirds of the Web survey estimates were significantly different from the Natsal-3 CASI estimates. Because the visually presented self-administered modes (ie, Web and CASI) do not yield similar estimates for the majority of questions, it appears that differences in mode (ie, measurement error) cannot fully explain the differences in the estimates between the Web surveys and Natsal-3, and that selection biases must also be present.

Of the 4 Web surveys, it was not possible to identify one that performed consistently better than the others, either over all estimates or in groups defined by survey mode in Natsal-3 (CAPI vs CASI) or question type (behavior vs opinions). This lack of predictability in how the Web surveys compare across estimates is consistent with findings for less sensitive measures in the United States [15,16].

Two of the Web surveys included additional quota controls known to be related to key estimates, but this did not lead to any consistent improvement. Although the modified quota Web surveys showed improved estimates for some variables, others did not change and yet others moved further away from the benchmarks. Moreover, the modified quotas presented operational difficulties for the 2 agencies carrying out the Web surveys and had to be relaxed in both cases.

Consistent with findings reported elsewhere that participants answering opinion questions in a self-administered mode (eg, on the Web or in CASI) are more likely to “satisfice” (ie, make less cognitive effort to think about and answer a survey question) than are those to a personal interview [13,19,35,36], the Web surveys and Natsal-3 CASI showed much greater use of neutral points (ie, “don’t know,” “depends,” or “neither agree nor disagree”) when compared with the same (or similar) opinion questions in Natsal-3 CAPI (Multimedia Appendix 7).

Although only a shortened version of the Natsal-3 questionnaire was included in the Web surveys, the questions were included in the same order as in Natsal-3 and were identically worded. Many of the questions excluded from the Web surveys had to do with the participant’s health condition and were aimed at the older age group (45-74 years) in Natsal-3 but who were not included in the Web survey sample. Also, in this paper, we have only looked at overall differences in prevalence estimates for men and women, and not at any other subgroup analysis and we have not looked at relationships among variables, but this analysis is planned for a subsequent paper. In further work, we shall also examine the degree to which other weighting/adjustment can reduce the bias seen here in the Web surveys.

Finally, we were not able to compare Natsal-3 results with those from a Web panel selected using probability sampling methods because no such panels have been recruited in Britain. An interesting future study would be to compare results between Natsal-3 and an Internet survey recruited using probability sampling methods, although the limited evidence available elsewhere suggests that the Internet survey results are still likely to differ significantly from those using face-to-face interviewing methods [37].

The demand for Web surveys, including in the field of health and epidemiological surveys, is likely to continue to increase as researchers look for new methods of data collection that are cost-effective while maintaining scientific rigor [38]. Commissioning Web surveys among the volunteer panels maintained by large market research agencies is a possible route because they are able to provide a quick turnaround at a much lower cost than traditional interview methods. Although using these volunteer Web panels may be suitable for certain types of study—potentially including surveys of hard-to-reach groups [4], for testing the properties of psychometric questionnaires [14], for syndromic surveillance [39], or for epidemiological studies where representative sampling may not be required [40]—the evidence from our investigation within Britain supports the conclusion that such surveys are not appropriate substitutes for probability-based sample surveys that aim to provide scientifically robust population estimates.

## Multimedia Appendix 1

Natsal-3 questionnaire content.

## Multimedia Appendix 2

Natsal Web questionnaire.

## Multimedia Appendix 3

Behavioral variables: 4 Web surveys compared with Natsal-3, separately for men and women.

## Multimedia Appendix 4

Achieved and target quotas for the modified quota Web surveys.

## Multimedia Appendix 5

Participant characteristics: 4 Web surveys and Natsal-3 compared with independent benchmarks, separately for men and women.

## Multimedia Appendix 6

ORs (95% CIs) for participant characteristics: 4 Web surveys and Natsal-3 compared with independent benchmarks, separately for men and women.

## Multimedia Appendix 7

Opinion variables: 4 Web surveys compared with Natsal-3, separately for men and women.

## Multimedia Appendix 8

ORs (95% CIs) for behavioral and opinion variables: 4 Web surveys compared with Natsal-3, separately for men and women.

## Abbreviations

CAPI: computer-assisted personal interview
CASI: computer-assisted self-interview
IHS: Integrated Household Survey
IMD: Index of Multiple Deprivation
LISS: Longitudinal Internet Study for the Social sciences
Natsal-3: Third National Survey of Sexual Attitudes and Lifestyles
ONS: Office for National Statistics
