# Supplementary material for: Nonprobability Web Surveys to Measure Sexual Behaviors and Attitudes in the General Population: A Comparison With a Probability Sample Interview Survey
Source: J Med Internet Res. 2014 Dec 8;16(12):e276. doi: 10.2196/jmir.3382 (PMC4275497; doi:10.2196/jmir.3382)
Supplement: Supplementary file 1 [file jmir_v16i12e276_app1.pdf]

**Questionnaire topic**	**CAPI**	**CASI**
General health, health conditions, medications taken, medical procedures (that may affect a person’s sex life)	X	
Family when growing up	X	
Learning about sex	X	
First heterosexual experience	X	
Contraception used	X	
Periods, menopause and use of hormone replacement therapy	X	
Experience of different heterosexual practices (vaginal, oral and anal intercourse)		X
Opposite-sex sex in the last 4 weeks and condom use		X
Same-sex sexual experiences (types of sexual practices, sex in last 4 weeks)		X
Number of opposite-sex partners in different time periods (lifetime, 5 years, 1 year, 3 months)		X
Number of same-sex partners in different time periods		X
Details of most recent partners		X
Having sex with people from other countries and while abroad		X
Non-volitional sex		X
Paying for sex		X
Family formation, pregnancy history and unplanned pregnancy		X
Fertility intentions and infertility		X
STI diagnoses and clinic attendance, HPV vaccination and cervical screening		X
Circumcision		X
HIV testing		X
Sexual function and satisfaction		X
Use of Viagra		X
Use of recreational drugs		X
Screen for depressive symptoms		X
Attitudes to different kinds of relationship and sexual lifestyles	X	
Perceived risk of HIV and other STIs	X	
Previous live-in partnerships	X	

Socio-demographics	X

The web surveys did not include all the questionnaire topics covered by Natsal-3 and, for some topics, included only some of the questions used in Natsal-3.
