# Supplementary material for: Nonprobability Web Surveys to Measure Sexual Behaviors and Attitudes in the General Population: A Comparison With a Probability Sample Interview Survey
Source: J Med Internet Res. 2014 Dec 8;16(12):e276. doi: 10.2196/jmir.3382 (PMC4275497; doi:10.2196/jmir.3382)
Supplement: Supplementary file 2 [file jmir_v16i12e276_app2.pdf]

NATSAL WEB QUESTIONNAIRE NatCen v1

THE NATIONAL SURVEY OF SEXUAL ATTITUDES AND LIFESTYLES (Natsal)

NATSAL – WEB COMPARISON QUESTIONNAIRE

COMPUTER QUESTION LISTINGS

Outline of questionnaire

GENERAL HEALTH....3
FAMILY 7
LEARNING ABOUT SEX....8
Periods and menopause....10
FIRST SEXUAL EXPERIENCES....11
SEXUAL ATTRACTION AND EXPERIENCE 15
CALCULATE DMDEBUT FOR FILTERING AT NEXT QUESTION:....15
IF FIrstint IN[13..44] then....15
IF XFrstint IN[13..44] then....15
IF (Firstint IN[1..12]) or IF (XFrstint IN[1..12]) then....15
nextint....15
IF NEXT AGE IN[13..44] then....15
sexualterms....17
HETEROSEXUAL SEX 18
HOMOSEXUAL SEX....21
NUMBER OF PARTNERS....26
PAYING FOR SEX....32
FAMILY FORMATION....33
SEXUALLY TRANSMITTED INFECTIONS AND HPV VACCINATIONS....35
HIV TESTING....38
SEXUAL FUNCTION 39
DRUGS....48

GENERAL HEALTH

1. Info1

Natsal 2012 is a national study of health and relationships that takes place every 10 years across Britain. It is funded by the Medical Research Council and the Wellcome Trust. We are asking you to take part in this important study online to help improve health education, family planning, and other health services that we may all need throughout our lives.

Your answers will be kept strictly confidential and no-one looking at the study findings will be able to identify you in any way.

This web study is being carried out by NatCen, University College London Medical School (UCL) and the London School of Hygiene and Tropical Medicine (LSHTM).

How will the study take place?

Please read the questions and to enter the answers online. The study asks some questions about relationships and experiences, and you can take part no matter what your background or experience is. You can skip any questions you do not want to answer in the questionnaire. You are also free to stop answering the questions at any time.

The questionnaire can take around 20 minutes to complete, but it may take less or more time for some people. Although it is best to fill in the questionnaire at one sitting, you also have the option to save your answers partway and return to the questionnaire at a later time.

You have already taken part in phase 1 of the study and we are now asking you to complete phase 2 by taking part in this study online.

More Information button

More information screen

Is the study confidential?

Yes. We take great care to protect the confidentiality of the information we are given. The study results will not be presented in a form which can reveal your identity.

Do I get anything from the study?

We will send you a £5 high street gift voucher as a token of our appreciation for taking part once you have completed the questionnaire. This may take up to four weeks to arrive.

Will I be asked the same questions as before?

In order to examine how people's behaviour and attitudes may change over time, and to evaluate how well an online version of Natsal may work, several of the questions are similar to those you may have already answered.

Who has reviewed the study?

The study has been reviewed by an independent group of people called a Research Ethics Committee based in Oxford, who assess studies like this to ensure the rights, safety, wellbeing and dignity of those who take part are protected.

What If I have any other questions?

We hope this information page answers the questions you may have, and that it shows the importance of the study. If you have any other questions or concerns about the study, please visit our main website at: www.natsal.org or call us on freephone 0800 783 5890.

Thank you very much for your help with this study.

2. Intro1
Some of these questions are quite personal, so you may prefer to complete the questionnaire somewhere private. If you come to any question that you do not want to answer, just press 'next page' and it will take you to the next question.

You might find it helpful to do the questionnaire in 'full screen view' so you can see all the answer options fully. To do this press 'F11'. You can press F11 again to switch back to normal screen view (this option works with most web browsers).

3. RAge
Firstly, what was your age last birthday?
Please type in the exact age in years

4. RSex
Are you male or female?
1 Male
2 Female

5. Health
"How is your health in general?
Would you say it is..."
1. "...very good,"
2. "...good,"
3. "...fair,"
4. "...bad,"
5. "or very bad?"

6. Drink
Now a few questions about drinking alcohol - that is if you drink.

Do you ever drink alcohol nowadays?

Textfill to appear for women only: If you do not drink alcohol at the moment because you are pregnant please select 'no'.

1. Yes
2. No

IF (Drink = Yes) THEN
7. DrinkOft
How often have you had an alcoholic drink of any kind during the last 12 months?

1. Five or more days a week
2. Three or four days a week
3. Once or twice a week
4. Once or twice a month
5. Once or twice in the last 12 months
6. Not at all in the last 12 months

IF (DrinkOft = 1-4) THEN
8. ManyAlc2
This picture shows what we mean by units of alcohol.
About how many units do you usually have on the days when you have any?
Please don't count special occasions?

1. One or two
2. Three or four
3. Five or six
4. More than six
5. I only drink on special occasions
6. **Other answer**

IF (DrinkOft = 1-4) THEN
9. DrinkNum
Again, this picture shows what we mean by units of alcohol.
How often do you have (***six/eight***) or more units on one occasion?

1. Never
2. Less than monthly
3. Monthly
4. Weekly

5. Daily or almost daily

END IF
END IF
END IF

ASK ALL
10. SmokeNow
Do you ever smoke cigarettes at all nowadays?
Include roll-ups but exclude cigars.
1. Yes
2. No

IF (SmokeNow = Yes) THEN
11. NoSmoke
About how many cigarettes a day do you usually smoke?

Enter a number per day.
If you can only think of a range, enter the middle number in that range.
If you smoke roll-ups, estimate the number of roll-ups.
If you smoke more at weekends than weekdays, enter the weekend number.
If less than one a day, enter 0.
Numeric: 0...97

IF (SmokeNow=No) THEN
12. ExSmoke
Did you ever smoke cigarettes regularly, that is, at least one cigarette a day?
1. Yes
2. No

END IF
END IF

FAMILY

13. BothMaPa
Now some questions about when you were growing up.

Did you live more or less continuously with **both** of your **natural** parents at home until you were 14?
Answer 'yes' if you were:

- at boarding school
- away temporarily
- or you had a parent serving in armed forces or away temporarily

1 Yes
2 No

IF (BothMaPa = No) THEN
14. WhoLive
Please look at the following list and say who you were living with when you were aged 14.
Stepfather/Stepmother includes cohabiting partners of natural parent.

1. Adoptive mother and father
2. Mother and stepfather
3. Father and stepmother
4. Mother only
5. Father only
6. In Local Authority care / foster home
7. Natural mother and father
8. Other (please say who at the next question)

IF (WhoLive=Other) THEN
15. XWhoLive
Please type in who you were living with when you were 14.
END IF

LEARNING ABOUT SEX

IF ((BothMaPa=Yes) OR (WhoLive=1, 2, 3, 4, 5 or 7)) THEN
16. TalkMaPA
When you were about 14, did you find it easy or difficult to talk to your (*textfill answer from wholive*) about sexual matters, or didn't you discuss sexual matters with (*them/her/him*) at that age?

You can only give one answer at this question.

1. Easy *(with one or both)*
2. Difficult
3. Didn't discuss (with either)
4. Varied/depended on topic

END IF

[Textfill is “parents” if bothmapa = yes, or from wholive “adoptive parents” (code 1) “mother” (code 2 or 4) or “father” (code 3 or 5) or “parents” (code 7)]

ASK ALL
17. LearnSex
When you were growing up, in which of the following ways did you learn about sexual matters?
You can select more than one answer at this question.

1. Mother (including step or adoptive)
2. Father (including step or adoptive)
3. Brother(s) / sister(s) (including half, step, or adoptive)
4. Lessons at school
5. Friends of about my own age
6. First (*girlfriend/boyfriend*) or sexual partner
7. Doctor, nurse or clinic
8. Television / radio / DVDs / videos
9. Books / magazines / newspapers
10. Internet – sexual advice websites
11. Internet – pornographic websites
12. Internet – other
13. Pornographic magazines / films
14. Other (please say what at next question)

IF (Other IN LearnSex) THEN
18. XLernSex
Please describe in what other ways you learned about sexual matters.
ENDIF

IF MORE THAN ONE ANSWER CODED AT LEARNSEX THEN
19. LernMost
From which one of these did you learn most?

1. Mother (including step or adoptive)
2. Father (including step or adoptive)
3. Brother(s) / sister(s) (including half, step or adoptive)
4. Lessons at school

5. Friends of about my own age
6. First (*girlfriend/boyfriend*) or sexual partner
7. doctor, nurse or clinic
8. Television / radio / DVDs / videos
9. Books / magazines / newspapers
10. Internet – sexual advice websites
11. Internet – pornographic websites
12. Internet – other
13. Pornographic magazines / films
14. (Other answer given at LearnSex)

END IF

PERIODS AND MENOPAUSE

IF (RSEX=female)
20. Periods2
How old were you when you started menstruating (having periods)?
Please type in the age in years. Please give an estimate if you cannot remember exactly.
If you have not started menstruating, type ‘97’.
Range 1...97
END IF

FIRST SEXUAL EXPERIENCES

ASK ALL

21.IntroInt

The next questions are about your own experience.

22. FirstInt

How old were you when you **first** had sexual intercourse with someone of the opposite sex, or hasn't this happened?
Please type in the exact age in years.
If you are not sure how old you were, type in '95' here and type in an estimate at the next question.
If this hasn't ever happened, type in '96'.
If you would rather not answer, type in '97'.
Range: 0...97

IF (FirstInt = 95) THEN

23. XFrstInt

Please type in the estimated age.
Range: 0...74

IF (FirstInt or XfrstInt <13) THEN

24. NextInt

Have you had sexual intercourse with anybody **else** of the opposite sex since you turned 13?

1 Yes
2 Not with anybody else since age 13
97 Would rather not answer

IF (NextInt = Yes) THEN

25. NextAge

How old were you then?
Please type in the exact age in years.
If you are not sure how old you were, type in '95' here and type in an estimate at the next question.
If you would rather not answer, type in '97'.
Range: 13...97

IF (NextAge = 95) THEN

26. XNxtAge

Please type in the estimated age.
Range: 13...74

END IF
END IF
END IF
END IF

ASK ALL
27. FirstExp
How old were you when you **first** had any type of experience of a sexual kind - for example, kissing, petting, or feeling one another - with someone of the opposite sex (or hasn't this happened either)?
Please type in the exact age in years.
If you are not sure how old you were, type in ‘95’ here and type in an estimate at next question.
If this hasn't ever happened, type in ‘96’.
If you would rather not answer, type in ‘97’.
Range: 0...97

IF (FirstExp = 95) THEN
28. XFrstExp
Please type in the estimated age.
Range: 0...74
END IF

IF ((Firstint or XFrstint or NextAge or XNxtAge >=13) AND FirstInt<>96,97) THEN
29. Age1Part
The next few questions are about the **first** time you had sexual intercourse with someone of the opposite sex (that is, the first person you had sex with after you turned 13).
How old was that partner at that time?
Please type in their age in years.
Please estimate their age if you can’t say exactly.
If you never knew their age, type in 97
If you can't remember their age, type in 98
Range: 1...98

30. Parts1st
As far as you now know, was it (also) (his/her) first time ever, or not?
If you don’t know, do you think it was (her/his) first time, or not?

31. BothWill
Would you say that you were both equally willing to have intercourse that first time, or was one of you more willing than the other?
1. Both equally willing
2. I was more willing
3. (He/She) was more willing

IF (BothWill = Partner more willing) THEN
32. PrtWill
Would you say that...
1 ... you were also willing,
2 or, that you had to be persuaded,
3 or, that you were forced?
END IF

IF (Bothwill=equally willing OR respondent more willing) OR (PrtWill=also willing OR persuaded) THEN

33. AnyPre2

Did you or your partner use any form of contraception or take any precautions that first time, or not?

You can select more than one answer at this question.

1 Condom (Sheath/Durex)
2 The pill
3 Emergency contraception
4 Other contraception
5 (*partner/I*) withdrew
6 Made sure it was a safe period
7 No precautions by me, don't know about partner
8 No precautions by either of us

END IF

34. JustMet2

Which one of these descriptions applies best to you and your partner at the time you first had intercourse?

Please select one answer only.

1. We had just met for the first time
2. We had recently met
3. We had known each other for a while, but were not in a steady relationship
4. We used to be in a steady relationship, but were not at that time
5. We were in a steady relationship at the time
6. We were living together as a couple / married at the time

35. RtTime

Looking back now to the first time you had sexual intercourse, do you think...

1 ...you should have waited longer before having sex with anyone,
2 or, that you should not have waited so long,
3 or, was it at about the right time?

IF (JustMet2 <> Married) THEN

36. FstRes

Which of these things applied to you **at the time**?

You can select more than one answer at this question.

1 I was curious about what it would be like
2 I was carried away by my feelings
3 Most people in my age group seemed to be doing it
4 It seemed like a natural 'follow on' in the relationship
5 I was a bit drunk at the time
6 I had smoked some cannabis
7 I had taken some other drugs
8 I wanted to lose my virginity
9 I was in love
10 Other particular factor (please say what at the next question)
11 Can't remember

IF FstRes=Other THEN
37. XFstRes
Please type in the other thing that applied at the time.
ENDIF
ENDIF

IF more than one answer coded at FstRes THEN
38. MainRea2
Please choose the **main** one that applied at the time.
1 I was curious about what it would be like
2 I was carried away by my feelings
3 Most people in my age group seemed to be doing it
4 It seemed like a natural 'follow on' in the relationship
5 I was a bit drunk at the time
6 I had smoked some cannabis
7 I had taken some other drugs
8 I wanted to lose my virginity
9 I was in love
10 Other particular factor
11 Can't choose one
12 Can't remember
ENDIF

SEXUAL ATTRACTION AND EXPERIENCE

ASK ALL

39. AttScale

Now please read the following answer list carefully as it is important that you understand it and are as honest as you can be in your answer. After you have read the list please select your answer.

I have felt **sexually attracted**...

1 Only to (*females/males*), never to (*males/females*)
2 More often to *(females/males)*, and at least once to a (*male/female*)
3 About equally often to *(females/males)* and to (*males/females*)
4 More often to (*males/females*), and at least once to a (*female/male*)
5 Only ever to *(males/females),* never to *(females/males)*
6 I have never felt sexually attracted to anyone at all
7 I would rather not say

40. ExpScale

As before, please read the following answer list carefully and be as honest as you can be in your answer. After you have read the list please select your answer.

Sexual experience is any kind of contact with another person that you felt was sexual (it could be just kissing or touching, or intercourse or any other form of sex). I have had some **sexual experience**...

1 Only with *(females/males)* (or a (*female/male*)), never with a (*male/female*)
2 More often with *(females/males)*, and at least once with a (*male/female*)
3 About equally often with *(females/males)* and with (*males/females*)
4 More often with (*males/females)*, and at least once with a (*female/male*)
5 Only with (*males/females*) (or a (*male/female*)), never with a (*female/male*)
6 I have never had any sexual experience with anyone at all
7 I would rather not say

CALCULATE DMDEBUT FOR FILTERING AT NEXT QUESTION:

IF FIRSTINT IN[13..44] THEN
DMDebut:=Firstint
ENDIF
IF FIRSTINT=95 THEN
XFRSTINT
IF XFRSTINT IN[13..44] THEN
DMDebut:=XFrstint
ENDIF
ENDIF
IF (FIRSTINT IN[1..12]) OR IF (XFRSTINT IN[1..12]) THEN
NEXTINT
IF NEXTINT = YES THEN
NEXT AGE
IF NEXT AGE IN[13..44] THEN
DMDebut:=NEXTAGE
ENDIF
IF NEXTAGE = 95 THEN
XNXTAGE
IF XNXTAGE IN [13..44] THEN

```
   DMDebut:=XNXTAGE
   ENDIF
ENDIF
```

SEXUALTERMS
IF (ExpScale IN 1 to 5, 7) OR (DMDebut >= 13) THEN
41. TermInt
Some of the questions that follow use terms like oral sex and vaginal intercourse. So that everyone attaches the same meaning to these terms, they are explained on the next two screens. Please be sure to read these explanations.

When you have finished reading each screen press <1> and <enter> to move on to the next one.

42. Terms1
PARTNERS OR SEXUAL PARTNERS:
People who have had sex together - whether just once, or a few times, or as regular partners, or as married partners.

GENITAL AREA:
A man's penis or a woman's vagina - that is, the sex organs.

VAGINAL SEX (vaginal sexual intercourse):
A man's penis in a woman's vagina.

ORAL SEX (oral sexual intercourse):
A (*woman/man'*s) or a (*man/woman*'s) mouth on a partner's genital area.

ANAL SEX (anal sexual intercourse):
A man's penis in a partner's anus (rectum or back passage).

Press <1> and <Enter> to continue

43. Terms2
SEXUAL INTERCOURSE OR 'HAVING SEX':
This includes vaginal, oral and anal sexual intercourse.

GENITAL CONTACT **NOT** INVOLVING INTERCOURSE:
Forms of contact with the genital area NOT leading to intercourse (vaginal, oral, or anal), but intended to achieve orgasm, for example, stimulating by hand (mutual masturbation).

MASTURBATION:
To arouse yourself sexually.

Press <1> and <Enter> to continue

HETEROSEXUAL SEX

44. LastVag

The next few questions are about different kinds of sex with *(WOMEN/MEN)*.
When, if ever, was the last occasion you had **VAGINAL SEXUAL INTERCOURSE** with a (*woman/man*)?
Vaginal sexual intercourse is a man's penis in a woman's vagina
If it has never happened, type in ‘7’.

1. In the last 7 days
2. Between 7 days and 4 weeks ago
3. Between 4 weeks and 6 months ago
4. Between 6 months and 1 year ago
5. Between 1 and 5 years ago
6. Longer than 5 years ago
7. Never had vaginal intercourse

45. OralYou

When, if ever, was the last occasion you had **oral sex** with a (*woman/man*) - by you to *(her/him)*, that is your mouth on *(her/his)* genital area?
If it has never happened, type in ‘7’.

1. In the last 7 days
2. Between 7 days and 4 weeks ago
3. Between 4 weeks and 6 months ago
4. Between 6 months and 1 year ago
5. Between 1 and 5 years ago
6. Longer than 5 years ago
7. Never had oral sex - by me to *(woman/man)*

46. OralPrt

When, if ever, was the last occasion you had **oral sex** with a (*woman/man*) - by *(her/him)* to you, that is *(her/his)* mouth on your genital area?
If it has never happened, type in ‘7’.

1. In the last 7 days
2. Between 7 days and 4 weeks ago
3. Between 4 weeks and 6 months ago
4. Between 6 months and 1 year ago
5. Between 1 and 5 years ago
6. Longer than 5 years ago
7. Never had oral sex - by *(a woman/man)** to me

47. AnalSex

When, if ever, was the last occasion you had **anal sex** with a (*woman/man*)?
Anal sex (anal sexual intercourse) is a man's penis in a partner's anus (rectum or back passage).
If it has never happened, type in '7'.

1. In the last 7 days
2. Between 7 days and 4 weeks ago
3. Between 4 weeks and 6 months ago
4. Between 6 months and 1 year ago
5. Between 1 and 5 years ago

6. Longer than 5 years ago
7. Never had anal sex

IF (any of OralYou, OralPart, lastvag, analsex in the last 4 weeks) THEN
48. Sex4Wks
On how many **occasions** in the last **4 weeks** have you had sex with a (*woman/man*)?
This means vaginal intercourse, oral sex, anal sex.
Click on the info button below if you want information about the meaning of these terms.
Please type in the number of occasions in the last **4 weeks**.
Please give an estimate if you can't say exactly.
Range: 0...200

IF (Sex4Wks>0) THEN
49. No4Wks
How many *(women/men)* have you had sex with in the last 4 weeks?
Please type in the number.
Range: 1...997

IF (No4Wks = 1) THEN
50. N1In4Wk
Was this a new partner with whom you had not had sex before?
1. Yes
2. No

ENDIF
ENDIF
ENDIF

IF (No4Wks > 1) THEN
51. New4Wks
How many of these were new partners with whom you had not had sex before?
Please type in the number.
Please type '0' if none.
Range: 0...997
ENDIF

IF (lastvag=in the last 4 weeks or analsex=in the last 4 weeks AND sex4wks>0) THEN
52. Cond4Wk
(*Did you use a condom (sheath) / Was a condom (sheath) used*) when having vaginal (or anal) sex with a (*woman/man*) in the last 4 weeks?
1. Yes, used every time
2. Yes, used sometimes
3. No, not used in the last 4 weeks
4. Not had vaginal (or anal) sex in last 4 weeks

ENDIF
ENDIF

IF (LastVag IN [WEEK..YEAR] OR AnalSex IN [WEEK..YEAR]) THEN

53. YrCond

In the last **YEAR** have you **ever** had **vaginal (*or anal*) intercourse** with a (*woman/man*) without using a condom?

Click on the info button below if you want information about the meaning of these terms.

1. Yes (have had intercourse without a condom in the past year)
2. No (have used a condom on all occasions of vaginal (*or anal*) intercourse in the past year)

IF (YrCond = Yes) THEN

54. NoNoCon

How many *(women/men)* have you had vaginal (*or anal*) intercourse with in the past year without using a condom?

Please type in the number.

Click on the info button below if you want information about the meaning of these terms.

Range: 1...997

ENDIF

ENDIF

HOMOSEXUAL SEX

55. EverSam
The next questions are about sex with (*men/women*)

Have you ever had **any** kind of sexual experience or sexual contact with a (*man/woman*)?
Please say 'yes' here, even if it was a long time ago or did **not** involve contact with the *(genital area/penis/vagina)*.
Click on the info button below if you want information about the meaning of this term.
1. Yes
2. No

[Note: Textfill for men is “genital area/penis” and for women “genital area/vagina”.]

IF (EverSam = Yes) THEN
56. FrstSam
How old were you the first time that happened?
Please type in the age in years.
Range: 1..45

57. GeniSam
Have you had sex with a (*man/woman*) involving *(genital area/penis/vaginal)* contact?
(That is oral (*or anal*) sex or any other contact involving the genital area.)
Click on the info button below if you want information about the meaning of this term.
1. Yes
2. No

IF (GeniSam = Yes) THEN
58. AgeGSam
And how old were you the first time you had sex with a (*man/woman*) involving *(genital area/penis/vaginal)* contact?
This could be the same age you just gave, or older.
Please type in the age in years
Range: 1...45

IF (AgeGSam < 13) THEN
59. NextAgeG
Have you had sex with any other *(men/women)* involving *(genital area/penis/vaginal)* contact since you turned 13?
1. Yes
2. Not with anybody else since age 13

IF (NextAgeG = Yes) THEN
60. NextASam
How old were you then?
Please estimate how old you were if you can’t say exactly. If you are unable to estimate please type in ‘97’."

Range: 13...97
ENDIF
ENDIF
ENDIF
ENDIF

IF (AgeGSam >12 OR NextAgeG = Yes) THEN
61. SamOYou
This is about different kinds of sex with (*male/female*) partners, involving contact with the *(genital area/penis/vagina)*.
When, if ever, was the last occasion you had **oral sex** with a (*man/woman*) - **by you to*(him/her)***, that is your mouth on *(his/her)* genital area?

1. In the last 7 days
2. Between 7 days and 4 weeks ago
3. Between 4 weeks and 6 months ago
4. Between 6 months and 1 year ago
5. Between 1 and 5 years ago
6. Longer than 5 years ago
7. Never had oral sex - by me to a *(man/woman)*

62. SamOPar
When, if ever, was the last occasion you had **oral sex** with a (*man/woman*) - **by *(her/him)* to you**, that is *(his/her)* mouth on your genital area?

1. In the last 7 days
2. Between 7 days and 4 weeks ago
3. Between 4 weeks and 6 months ago
4. Between 6 months and 1 year ago
5. Between 1 and 5 years ago
6. Longer than 5 years ago
7. Never had oral sex - by a *(man/woman)* to me

IF (RSex = Male) THEN
63. SamAYou
When, if ever, was the last occasion you had **anal sex** with a man - **by you to him**?
That is your penis in his anus (rectum or back passage).

1. In the last 7 days
2. Between 7 days and 4 weeks ago
3. Between 4 weeks and 6 months ago
4. Between 6 months and 1 year ago
5. Between 1 and 5 years ago
6. Longer than 5 years ago
7. Never had anal sex - by me to a man

64. SamAHim

When, if ever, was the last occasion you had **anal sex** with a man - **by him to you**?
That is his penis in your anus (rectum or back passage).

1. In the last 7 days
2. Between 7 days and 4 weeks ago
3. Between 4 weeks and 6 months ago
4. Between 6 months and 1 year ago
5. Between 1 and 5 years ago
6. Longer than 5 years ago
7. Never had anal sex - by a man to me

ENDIF

65. SamGen
When was the last occasion you had any **other form of sex** with a (*man/woman*) that involved genital contact but **not** oral (*or anal)* sex?
Genital contact NOT involving intercourse is forms of contact with the genital area NOT leading to oral (*or anal*) intercourse, but intending to achieve orgasm, for example, by stimulating by hand.
Click on the info button below if you want information about the meaning of other terms.

1. In the last 7 days
2. Between 7 days and 4 weeks ago
3. Between 4 weeks and 6 months ago
4. Between 6 months and 1 year ago
5. Between 1 and 5 years ago
6. Longer than 5 years ago
7. Never had genital contact without oral and/or anal sex as well

IF ((AgeGSam>12 OR NextAgeG =Yes) AND (any of samoyou, samopar, samayou, samahim in last 4 weeks)) THEN
66. Sam4Wks
On how many occasions in the last **4 weeks** have you had sex with a (*man/woman*)?
Please give an estimate if you can't say exactly.
Please type in the number, or ‘0’ if none.
Range: 0...200

IF (Sam4wks>0) AND (RSex = Male) AND (samahim = in last 4 weeks OR samayou = in last 4 weeks) THEN
67. SamCo4W
Was a condom (sheath) used on any occasions of having anal sex with a man in the last 4 weeks?

1. Yes, used on every occasion
2. Yes, used on some occasions
3. No, not used in the last 4 weeks
4. Not had anal sex in the last 4 weeks

ENDIF
ENDIF

IF (SamAYou IN [WEEK..YEAR] OR SamAHim IN [WEEK..YEAR]) THEN
68. AnCom
In the last year, when you've had anal sex, how often have you, or your partner, used a condom?
Click on the info button below if you want information about the meaning of the term ‘anal sex’.

1 Every time
2 Most of the time
3 Occasionally
4 Not at all in the last year

IF AnCom IN [Most..None] THEN
69. NoAnCom
In the last year, with how many men have you had anal intercourse without using a condom?
Click on the info button below if you want information about the meaning of the term ‘anal sex’.
Please type in the number, or ‘0’ if none.
Range: 0..997
ENDIF
ENDIF

IF (AgeGSam >12 OR NextAgeG = Yes) AND (RSex=male) THEN
70. GayPub
How often, if at all, do you usually go to gay pubs, bars, or clubs?

1. At least once a week
2. Less often but at least once a month
3. Less often but at least twice a year
4. Less often but at least once a year
5. Less often than once a year
6. Never

ENDIF

Derive “AskWEB”
1 = Full
2 = Short
Set default for AskWeb=1 (Full).
IF (DMDebut < 13 OR FirstInt IN [96, 97]) AND (lastvag to analsex all = 7) AND ((evesam=2 or genisam=2) or (GeniSam = Yes AND AgeGSam < 13 AND NextAgeG = No))

AskWeb=2 (Short)

NUMBER OF PARTNERS

IF ((any of lastvag, oralyou, oralprt, analsex < 7) OR (genisam=yes AND (agegsam>12 or nextasam>12))) AND AskWEB=Full THEN

71. PartInt

The next questions are about the number of people you have had sex with at different times in your life.

When answering these questions please include everyone you have ever had sex with, whether it was just once, a few times, a regular partner or (*wife/husband*).
Be as accurate as you can: give your best estimate if you can't remember exactly.

IF (any of lastvag, oralyou, oralprt, analsex < 7 OR DK OR Ref) THEN

72. HetLife

Altogether, in your life so far, how many (***women/men***) have you had sexual intercourse with (vaginal, oral or anal)?
Please type in the number, or '0' if none.
Click on the info button below if you want information about the meaning of these terms.
Range: 0...9997

IF (HetLife > 4) THEN

73. HetCalc

Which of these best describes how you worked out that answer?

1. I just knew the number
2. I remembered each partner, and counted them up
3. I estimated or guessed the number
4. I remembered some partners and then added on an estimated number for others
5. Other

END IF
END IF
END IF

IF (HetLife > 0 OR DK) THEN

74. Het5Yrs

Altogether, in the last **5 years**, how many (*women/men*) have you had sexual intercourse with?
Please type in the number, or '0' if none.
Click on the info button below if you want information about the meaning of these terms.
Range: 0...9997

IF Het5yrs>0 THEN

75. Het1Yr

And altogether, in the **last year**, how many (*women/men*) have you had sexual intercourse with?
Please type in the number, or '0' if none.

Click on the info button below if you want information about the meaning of these terms.
Range: 0...997

IF (Het1Yr > 1) THEN
76. NoNewPt
How many of these (*women/men*) were new partners who you had sex with for the first time during the last year?
Please type in the number, or '0' if none.
Range: 0...997

ELSEIF (Het1Yr = 1) THEN
77. HetNewP
Was this (*woman/man*) a new partner who you had sex with for the first time during the last year?

1. Yes
2. No

ENDIF
ENDIF
ENDIF
ENDIF

Same sex partners

IF (genisam=yes AND (agegsam>12 or nextasam>12)) THEN

78. SamLife

Altogether, in your life so far, how many **(*men/women*)** have you had sex with (that is oral *or anal* sex *or* other forms of genital contact)?
Please type in the number, or '0' if none.
Click on the info button below if you want information about the meaning of these terms.
Range: 0...9997

IF (SamLife > 4) THEN

79. SamCalc

Which of these best describes how you worked out that answer?

1. I just knew the number
2. I remembered each partner, and counted them up
3. I estimated or guessed the number
4. I remembered some partners and then added on an estimated number for others
5. Other

END IF

END IF

IF (SamLife > 0 OR DK) THEN

80. Sam5Yrs

Altogether, in the last **5 years,** how many (*men/women*) have you had sex with?
Please type in the number, or '0' if none.
Range: 0...9997

IF (Sam5Yrs > 0) THEN

81. Sam1Yr

And - altogether, in the **last year**, how many (*men/women*) have you had sex with?
Please type in the number, or '0' if none.
Range: 0...997

IF (Sam1Yr > 1) THEN

82. HNoNewP

How many of these (*men/women*) were new partners who you had sex with for the first time during the last year?
Please type in the number, or '0' if none.
Range: 0...997

ELSEIF (Sam1Yr = 1) THEN

83. SamNewP

Was this (*man/woman*) a new partner who you had sex with for the first time during the last year?

1 Yes
2 No

ENDIF
ENDIF
ENDIF

Ask all who report more than one partner (same sex+opposite sex) in the last 5 years:
IF (Het5Yrs + Sam5Yrs >1) THEN

84. Overlp5y

Thinking about **all** of the people you have had sex with in the last five years, did any of them overlap in time? In other words did you have sex with someone (person A), then have sex with someone else (person B), and then have sex with the first person (person A) again.

1. Yes
2. No

ENDIF

SEX ABROAD

IF (Het5Yrs > 0) OR (Sam5Yrs > 0) THEN

85. TravInt

The next few questions are about having sex when **visiting** countries outside the UK (i.e. outside of England, Scotland, Wales and Northern Ireland).

86. Travel

In the last 5 years, have you traveled outside the UK, for any reason?

1. Yes
2. No

IF (Travel = Yes) THEN

87. TrvlSex

And, in the last 5 years, have you had sex with anyone **for the first time** while you were in any country outside the UK?

Include **UK citizens** and/or **others** you **first** had sex with while in another country.

1. Yes
2. No

IF (TrvlSex = Yes) THEN

88. NoSexTl

In the last 5 years, how many people did you have sex with **for the first time** while you were in any country outside the UK?

Please type in the number.

Include **UK citizens** and/or **others** you **first** had sex with while abroad.

Range: 1...9997

89. Cntry1

And where did *(this/these)* new partner*(s)* normally live?

Please note: we are interested in which country the person normally lives, not the country where you met.

Select the region that covers each country. You can select more than one region at this question.

1 UK (but first had sex with them while abroad)
2. Other European countries (including Ireland, Eastern Europe, Russia)
3. Australia, New Zealand
4. North America (USA and Canada)
5. South America, Central America (including Mexico)
6. Caribbean countries
7. Asian countries (including China, India, Pakistan, Bangladesh, Thailand, Malaysia, etc)
8. Middle East, North Africa
9. African countries (other than North Africa)
10. Other region or country (please say which at the next question)

11. Don't know which region or country

IF (Other IN Cntry1) THEN

90. OthCntr1

Please type in the name of the region or country.

Text: Maximum: 60 characters

ENDIF

ENDIF

ENDIF

91. SexAbrd

This question is about people you may have had sex with here in the UK, but who normally live in another country.

In the last **5 years**, have you had sex for the first time, here in the UK, with anyone who normally lives OUTSIDE the UK?

Include anyone who was visiting the UK, or living here for a while.

1. Yes
2. No

ENDIF

PAYING FOR SEX

IF (HetLife>=0 OR SamLife>=0) AND AskWEB=Full THEN
92. EverPd
The next questions are about paying for sex.

Have you ever paid money for sex with a *man/woman*?"
1. Yes
2. No

IF (EverPd=Yes) THEN
93. LastPay
When was the last time you paid money for sex with a *man/woman*?
1. In the last 7 days
2. Between 7 days and 4 weeks ago
3. Between 4 weeks and 1 year ago
4. Between 1 year and 5 years ago
5. Longer than 5 years ago

END IF
END IF

IF (Rsex=male and eversam=yes) THEN
94. SamPaid
Have you ever paid money for sex with a man?
1. Yes
2. No

IF (SamPaid=Yes) THEN
95. SamPLst
When was the last time you paid money for sex with a man?
1. In the last 7 days
2. Between 7 days and 4 weeks ago
3. Between 4 weeks and 1 year agO
4. Between 1 year and 5 years ago
5. Longer than 5 years ago

ENDIF
ENDIF

FAMILY FORMATION

IF (lastvag = 1-6 OR DK OR Ref)
96. AnyChild
The next questions are about your family.
Do you have, or have you had, any children that you are the natural/biological parent of?

Please include any who don't now, or never did, live with you as part of your household.

Please include those who have died, but exclude adopted children, still born children, abortions or miscarriages.

1. Yes
2. No

IF AnyChild=Yes THEN
97. NoChild
How many children have you had?
Please include those who have died, but not still born children.
Numeric: 1...97

98. DoBChM
In which month and year was your first child born?
Please enter the month at this question and the year at the next question.

99. DoBChY
Please enter the year your first child was born.
Numeric: 1970.2012
END IF
END IF

IF (RSex**=**female AND **(**lastvag = 1-6 OR DK OR Ref))
100. Abort
Have you ever had a termination of pregnancy (abortion)?

1. Yes
2. No

IF (Abort = Yes) THEN
101. ManyAb
How many terminations of pregnancy (abortions) have you had?
Range: 1..20

IF (ManyAb = 1) THEN
102. AgeAb
What age were you then?
Please type in the age in years.
Range: 12..45

ELSEIF (ManyAb > 1) THEN
103. AgeAbFir
What age were you when you had the FIRST termination?
Please type in the age in years.
Range Range: 12..45

104. AgeAbLas
What age were you when you had the LAST one?
Please type in the age in years.
Range: 12..45
ENDIF
ENDIF
ENDIF
ENDIF

SEXUALLY TRANSMITTED INFECTIONS AND HPV VACCINATIONS

IF (HetLife>0 OR SamLife>0) AND AskWEB=Full THEN

105. STDClin

The next questions are about infections that can be transmitted by sex. Please answer even if you have never had an infection that was transmitted by sex.

Have you ever attended a sexual health clinic (GUM clinic)?

1. Yes
2. No

IF (STDClin=Yes) THEN

106. WhnClin

When was that?

If you have been more than once, please say the last time you went

1. Less than 1 year ago
2. Between 1 and 5 years ago
3. Between 5 and 10 years ago
4. More than 10 years ago

END IF

107. Diagnos

Have you ever been told by a doctor or other healthcare professional that you had any of the following?

Please select any that you have had, even if not transmitted by sex (women only: Thrush).

If more than one, press the space bar between each number.

If you have not had any, please select 'none of these' at the bottom of the list.

1. Chlamydia
2. Gonorrhoea
3. Genital warts (venereal warts)
4. Syphilis
5. Trichomonas vaginalis (Trich, TV)
6. Herpes (genital herpes)
7. Pubic lice / crabs
8. Hepatitis B
9. (Men only:) NSU (Non Specific Urethritis), NGU (Non Gonococcal Urethritis)
10. (Men only:) Epididymitis
11. (Women only:) Pelvic Inflammatory Disease (PID, salpingitis)
12. (Women only:) Vaginal thrush (Candida, Yeast infection)
13. (Women only:) Bacterial vaginosis
14. Yes, but can't remember which
15. None of these

IF (Diagnos=Chlamydia) THEN

108. WhnChlam

When were you last told by a doctor or healthcare professional that you had Chlamydia?

1. Less than 1 year ago
2. Between 1 and 5 years ago
3. Between 5 and 10 years ago
4. More than 10 years ago

END IF

IF (Diagnos=Gonorrhoea) THEN
109. WhnGono
When were you last told by a doctor or healthcare professional that you had Gonorrhea?
1. Less than 1 year ago
2. Between 1 and 5 years ago
3. Between 5 and 10 years ago
4. More than 10 years ago

END IF

IF (Diagnos=Genital warts) THEN
110. WhnWarts
When were you last told by a doctor or healthcare professional that you had Genital Warts (venereal warts)?
1. Less than 1 year ago
2. Between 1 and 5 years ago
3. Between 5 and 10 years ago
4. More than 10 years ago

END IF

IF (Diagnos=Syphilis) THEN
111. WhnSyphl
When were you last told by a doctor or healthcare professional that you had Syphilis?
1. Less than 1 year ago
2. Between 1 and 5 years ago
3. Between 5 and 10 years ago
4. More than 10 years ago

END IF

IF (Diagnos=Trich) THEN
112. WhnTric
When were you last told by a doctor or healthcare professional that you had Trichomonas vaginalis (trich, TV)?
1. Less than 1 year ago
2. Between 1 and 5 years ago
3. Between 5 and 10 years ago
4. More than 10 years ago

END IF

IF (Diagnos=Herpes) THEN

113. WhnHerp
When were you last told by a doctor or healthcare professional that you had Herpes (genital herpes)?
1. Less than 1 year ago
2. Between 1 and 5 years ago
3. Between 5 and 10 years ago
4. More than 10 years ago

END IF

IF (Diagnos=NSU) THEN
114. WhnNSU
When were you last told by a doctor or healthcare professional that you NSU (Non Specific Urethritis), NGU (Non Gonococcal Urethritis)?
1. Less than 1 year ago
2. Between 1 and 5 years ago
3. Between 5 and 10 years ago
4. More than 10 years ago

END IF

HIV TESTING

ASK ALL

115. HIVTstN

Have you ever had a blood test for HIV (the virus that causes AIDS)?

1. Yes
2. No
3. Maybe/Not sure

IF (HIVTstN=Yes)

116. WhenTest

When was that test?

If you have had more than one test, please say the when the most recent test was

1. In the last year
2. Between 1 and 2 years ago
3. Between 2 and 5 years ago
4. Longer than 5 years ago

END IF

SEXUAL FUNCTION

IF (Hetlife>0 OR Samlife>0) THEN

119. HafSex

The next few questions are about things that may affect your sexual activity and enjoyment.

In the last year, have you had any health condition or disability that you feel has affected your sexual activity or enjoyment in any way?

1 Yes
2 No

120. MafSex

Have you taken any medications in the last year that you feel have limited your sexual activity or enjoyment in any way?"

1 Yes
2 No

IF (het1yr>0 or sam1yr>0) THEN

121. SFDefn

The next few questions are about your sex life. Some questions use the term ‘having sex’. By this we mean vaginal, oral, or anal sexual intercourse.

122. SFPrb

Some people go through times when they are not interested in sex or find it difficult to enjoy sexual activities. The questions that follow are about some common difficulties that people experience.

In the last year, have you experienced any of the following **for a period of 3 months or longer?**

Please type in the number of every one that you have experienced **for a period of 3 months or longer.**

You can type in more than one number by pressing the spacebar between each number.

If you have not experienced any please type in ‘10’.

1. Lacked interest in having sex
2. Lacked enjoyment in sex
3. Felt anxious during sex
4. Felt physical pain as a result of sex
5. Felt no excitement or arousal during sex
6. Did not reach a climax (experience an orgasm) or took a long time to reach a climax despite feeling excited/aroused
7. Reached a climax (experienced an orgasm) more quickly than you would like
8. Had an uncomfortably dry vagina (asked of women only)
9. Had trouble getting or keeping an erection (asked of men only)
10. I did not experience any of these

IF (SFPrb =Any problems) THEN following loop is asked for each problem:

123. SFPrLng

You said that you ***(name of problem)***. For how long have you experienced / did you experience this?

1. At least 3 months but less than 6 months
2. At least 6 months but less than a year
3. At least a year but less than 5 years
4. 5 years or longer

124. SFPrFrq

You said that you *(name of problem)* for *(length of time at SFPrLng)*. Thinking about the times you had sex (or tried to have sex) during this period, how often did this occur?

1. Always
2. Very often
3. Sometimes
4. Not very often

125. [BUT IF SFPrb=1 (interest in sex) then use the following wording for SFprFrq:]

You said that you lacked interest in having sex for *(length of time given at SFPrLng)*.During this period, how often did you experience this?

1. Always
2. Very often
3. Sometimes
4. Not very often

126. SFPrbDs

And how do you feel about this?

1. Not at all distressed
2. A little distressed
3. Fairly distressed
4. Very distressed

END IF
END IF
END IF

ASK ALL
117. Ptnrst
At present are you…

1. Married and living with your (*husband/wife*)
2. In a registered same-sex civil partnership and living with your partner
3. Living with a partner, as a couple (not married or in a civil partnership)
4. In a steady relationship, but not living together
5. None of the above

IF (Ptnrst = 1-4) THEN
118. Ptnryrs

You said you are <textfill from Ptnrst>. For how long, in total, have you been with this partner?

1. Less than one year
2. At least one year but less than five
3. Five years or more

IF ((Ptnryrs=2 or 3) AND (het1yr>0 or sam1yr>0)) THEN

127. SFRInt

Thinking about your relationship with your partner in the last year, how much do you agree or disagree with the following statements:

My partner and I share about the same level of interest in having sex.

1. Agree strongly
2. Agree
3. Neither agree nor disagree
4. Disagree
5. Disagree strongly

128. SFRlikes

My partner and I share the same sexual likes and dislikes.

1. Agree strongly
2. Agree
3. Neither agree nor disagree
4. Disagree
5. Disagree strongly

129. SFRPDf

My partner has experienced sexual difficulties in the last year.

1. Agree strongly
2. Agree
3. Neither agree nor disagree
4. Disagree
5. Disagree strongly

130. SFREm

I feel emotionally close to my partner when we have sex together.

1. Always
2. Most of the time
3. Sometimes
4. Not very often
5. Hardly ever

131. RelSat
On a scale of 1 to 7, where 1 means very happy and 7 means very unhappy:
How happy or unhappy are you with your relationship with your partner, all things considered?
1..7

END IF
END IF

ASK ALL
132. SFFSatis
The next few questions ask about your sex life in the last year. An individual's sex life includes their sexual thoughts, sexual feelings, sexual activity and sexual relationships.

Thinking about your sex life in the last year, how much do you agree or disagree with the following statements:

I feel satisfied with my sex life
1. Agree strongly
2. Agree
3. Neither agree nor disagree
4. Disagree
5. Disagree strongly

133. SFFDst
I feel distressed or worried about my sex life.
1. Agree strongly
2. Agree
3. Neither agree nor disagree
4. Disagree
5. Disagree strongly

IF (Age>17) OR ((Age=(16-17) AND (lastvag=week..longer OR analsex=week..longer OR samahim=week..longer OR samayou=week..longer)) THEN
134. SFFavoid

I have avoided sex because of sexual difficulties, either my own or those of my partner.

1. Agree strongly
2. Agree
3. Neither agree nor disagree
4. Disagree
5. Disagree strongly

IF (SFavoid=1,2) THEN

135. SFavPrb

You said that you have avoided sex because of sexual difficulties. Please type in the number of every difficulty that caused you to avoid sex.

You can type in more than one number by pressing the spacebar between each number.

If you avoided sex because your partner experienced any of these difficulties type in ‘10’.

If none of these things caused you to avoid sex, please type in '11'.

1. Lacked interest in having sex
2. Lacked enjoyment in sex
3. Felt anxious during sex or felt anxious about having sex
4. Felt physical pain, or feared feeling physical pain as a result of sex
5. Felt no excitement or arousal during sex
6. Did not reach a climax (experience an orgasm) or took a long time to reach a climax despite feeling excited/aroused
7. Reached a climax (experienced an orgasm) more quickly than you would like
8. Had an uncomfortably dry vagina (asked of women only)
9. Had trouble getting or keeping an erection (asked of men only)
10. My partner had one (or more) sexual difficulty
11. None of these things caused me to avoid sex

END IF

END IF

136, SFHWch
Have you sought help or advice regarding your sex life from any of the following sources in the last year?

You can type in more than one number by pressing the spacebar between each number.

If you have not sought any help or advice, type in ‘11’.

1. Family member/friend
2. Information and support sites on the internet
3. Self-help books/Information leaflets
4. Self-help groups
5. Helpline
6. GP/Family doctor
7. Sexual health/GUM/STI clinic
8. Psychiatrist or psychologist
9. Relationship counsellor
10. Other type of clinic or doctor
11. Have not sought any help

IF Het1yr>0 OR sam1yr>0 THEN
137. Frequent
Thinking of the way things are for you these days, which one of these would you really prefer?"

1. "To have sex much more often than I do now"
2. “To have sex a bit more often"
3. "It is about right as it is"
4. "To have sex a little less often"
5. "To have sex much less often than I do now"

END IF

ATTITUDINAL QUESTIONS
ASK ALL
138. RskYou
There are different opinions about how many people are at risk of becoming infected with HIV, the virus that causes AIDS, but we would like to know what **you** think about the risks to **you**, personally, with your present sexual lifestyle? Do you think you are…

1. Greatly at risk
2. Quite a lot
3. Not very much
4. Not at all at risk
5. Don't know

139. STIRisk
People are also at risk of getting other sexually transmitted infections. What do you think about the risks to **you**, personally, with your present lifestyle of getting a sexually transmitted infection that is not HIV? Do you think you are…

1. Greatly at risk
2. Quite a lot
3. Not very much
4. Not at all at risk
5. Don't know

140. RWAdult
Now some questions on your views about different types of relationships.
Please tell us what your views are about the following sexual relationships.

A married person having sexual relations with someone other than his or her partner?

1. Always wrong
2. Mostly wrong
3. Sometimes wrong
4. Rarely wrong
5. Not wrong at all
6. Depends/Don't know

141. RWCasual
And what is your opinion about a person having one night stands?

1. Always wrong
2. Mostly wrong
3. Sometimes wrong
4. Rarely wrong
5. Not wrong at all
6. Depends/Don't know

142. RWSamm
What is your general opinion about sexual relations between two adult men?

1. Always wrong
2. Mostly wrong
3. Sometimes wrong

4. Rarely wrong
5. Not wrong at all
6. Depends/Don't know

143. RWSamf
And sexual relations between two adult women?

1. Always wrong
2. Mostly wrong
3. Sometimes wrong
4. Rarely wrong
5. Not wrong at all
6. Depends/Don't know

DRUGS

144. DrugUse
Have you **ever** taken any of the drugs listed below? (Please **do not** count any drugs you have injected.)
Please let us know **all** the drugs you have taken, but did not inject.
You can select more than one answer at this question.

If you have never taken any drugs, please select ‘none of these’ from the bottom of the list.

1. Cannabis (marijuana, grass, hash, ganja, draw, skunk, weed, spliff)
2. Amphetamines (speed, whizz, uppers, billy)
3. Cocaine or coke (charlie)
4. Crack (rock, stones, white)
5. Ecstasy (E)
6. Heroin that was not injected (smack, skag, H, brown, gear, horse)
7. Acid or LSD (tabs, trips) or magic mushrooms
8. Crystal Meth
9. Amyl Nitrates (poppers, liquid gold, rush)
10. Other non-prescribed drugs
11. None of these

IF (DrugUse = cannabis) THEN
145. DrCan12m
Have you taken cannabis in the last 12 months?
1. Yes
2. No

END IF

146. Inject2
Have you ever injected yourself with any **non-prescribed** drugs or other substances?
1. Yes
2. No

IF (Inject2=yes) THEN
147. WhenInj
When was the last time you injected yourself with **non-prescribed** drugs or other substances?
1. In the last 7 days
2. Between 7 days and 4 weeks ago
3. Between 4 weeks and 1 year ago
4. Over 1 year ago

IF (WhenInj=over 1 year ago) THEN
148. InjAgLst
How old were you the **last** time you injected yourself with **non-prescribed** drugs or other substances?
1...44

END IF

END IF **HOUSEHOLD CLASSIFICATION**
149. HHSize
Finally, a few questions about you and your household.
We would like to check who is living in your household at present.
Including yourself, how many people live here regularly as members of this household?
Type in the number of people.
Range: 1...14

IF (Ptnrst=3-5) THEN
MarStWeb
At present, are you…
1. **Single, that is never married and never registered in a same-sex civil partnership**
2. **Separated, but still legally married**
3. **Divorced**
4. **Widowed**
5. **Or something else? (please say what at the next question)**

MarOthWb
IF MarStWeb = other
Please type in what
END IF
END IF

IF (HHSize >1) THEN
150. HHWeb
How are the people living in your household related to you? You can select more than one option from the list.
1. Spouse/civil partner
2. Cohabiting partner
3. Son/daughter (incl. adopted)
4. Step-son/daughter
5. Foster child
6. Son-in-law/daughter-in-law
7. Parent/guardian
8. Step-parent
9. Foster parent
10. Parent-in-law
11. Brother/sister (incl. adopted)
12. Step- or half-brother/sister
13. Foster brother/sister
14. Brother/sister-in-law
15. Grand-child
16. Grand-parent
17. Other relative
18. Other non-relative (e.g. flat mates)

ENDIF

151. Acctype
What type of accommodation do you live in?
1 **Detached house**
2 **Semi-detached house**
3 **Terraced house (including end of terrace)**

4 Flat or maisonette – purpose-built
5 Flat or maisonette – conversion
6 Other

152. Tenure
Do you (or your household) own or rent this (house/flat/accommodation)?
1 Own it outright
2 Buying it with the help of a mortgage or loan
3 Pay part rent and part mortgage (shared ownership)
4 Rent it
5 Live here rent free (including rent free in relative's/friend's property; excluding squatting)

153. RActiv
Which of these descriptions applies to what you were doing last week, that is, in the seven days ending last Sunday?

You can select more than one answer.
1 Going to school or college full-time (including on vacation)
2 In paid employment or self-employed (or temporarily away)
3 On a Government scheme for employment training
4 Doing unpaid work for a business that you own, or that a relative owns
5 Waiting to take up paid work already obtained
6 Looking for paid work or a Government training scheme (unemployed)
7 Intending to look for work but prevented by temporary sickness or injury
8 Permanently unable to work because of long-term sickness or disability
9 Retired from paid work
10 Looking after home or family
11 Doing something else (Please say what at next question)

IF (Other IN RActiv) THEN
154. XRActiv
Please type what else you were doing last week..
END IF

155. TEdAge
At what age did you complete your continuous full-time education?
If you had a 'gap' year between school and university or college please include it as continuous.
If you have not yet finished, type in ‘96’.
Range: 1..96

156. ExamWeb
Please read down the following list and click on the highest qualification that you have, that is, the first one you come to.
1. Degree level qualification
2. A level
3. AS level
4. SCE/SLC/SUPE Higher / Advanced Higher / Higher / Intermediate / CSYS"

5. O-level, 1975 or earlier
6. O-level, after 1975 A-C
7. O-level, after 1975 D-E
8. GCSE grades A*-C
9. GCSE grades D-G
10. CSE grade 1, etc
11. CSE grades 2-5, etc
12. CSE Ungraded
13. SCE/SLC Standard / Ordinary (O) / Lower
14. SUPE Lower or Ordinary
15. School Certificate
16. Foreign qualification
17. None of these

157. Quals
Do you have any of the following qualifications?

You can select more than one answer.

1 Teaching qualification
2 Nursing qualification
3 HNC/HND, etc
4 ONC/OND, etc
5 City & Guilds Full
6 City & Guilds Advanced
7 City & Guilds Craft
8 NVQ Level 5
9 NVQ Level 4
10 NVQ Level 3, etc
11 NVQ Level 2, etc
12 NVQ Level 1, etc
13 Recognised trade apprenticeship completed
14 Clerical or Commercial Qual
15 Other vocational or professional qualification (please say what at next question)
16 None of these

IF (Other IN Quals) THEN
158. XQuals
Please type in the name of the other vocational or professional qualification.
END IF

IF (Age>16) THEN
159. Drvlc
Do you hold a full driving licence valid in Great Britain to drive a car?
1. Yes
2. No

160. Ethnic
To which of the following ethnic groups do you consider you belong?
A. White
1. British
2. Irish
3. Any Other White background

B. Mixed
4. White and Black Caribbean
5. White and Black African
6. White and Asian
7. Any Other Mixed background

C. Asian or Asian British
8. Indian
9. Pakistani
10. Bangladeshi
11. Any Other Asian background

D. Black or British Black
12. Caribbean
13. African
14. Any Other Black background

E. Chinese or other ethnic group
15. Chinese
16. Any Other

161. SexID
Which of the following options best describes how you think of yourself?
1. Heterosexual/Straight
2. Gay/Lesbian
3. Bisexual
4. Other

162. Comp1
You have now taken part in two phases of the study, the first involving a visit from an interviewer and the second completing a questionnaire over the web.
Would you say...
1. You answered more accurately when completing the first questionnaire with the interviewer
2. You answered more accurately when completing this web questionnaire
3. There was no difference in how accurately you answered the two questionnaires

163. Comp2
And did you feel more comfortable answering questions from this survey...
1. when completing the survey with the interviewer.
2. on the web,
3. or were you equally comfortable with both surveys?

163. Thank
Thank you very much for completing this questionnaire.

There are a range of organisations which provide professional and confidential help or advice on many of the topics raised in the questionnaire. Please click **here** for a list of some of these organisations and their websites.
List of helplines button

List of helplines:

Useful contact numbers and websites

There are many organisations which provide professional and confidential help or advice on a wide range of health and personal problems. The telephone numbers and websites of some of the main organisations are listed below. Any of them should be able to help or advise you on where to go locally for help or information.

NHS website

Provides information about sexual health and allows you to search for your nearest sexual health service

Web addresses: www.nhs.uk/livewell/sexualhealth

www.nhsdirect.nhs.uk

Your registered GP

Can provide consultation and referral to specialist agencies

You can find a GP on the **NHS website**:

www.nhs.uk

Family Planning Association

Provides guidance on where you can seek help for a wide range of issues relating to health, sexual and personal relationships

Web address: www.fpa.org.uk

Telephone: 0845 122 8690

Marie Stopes International

Provides advice and information on a range of topics including family planning, abortion, contraception, sexually transmitted diseases and menopause

Web address: www.mariestopes.org.uk

Email address: services@mariestopes.org.uk

Helpline: 0845 300 8090

Sexual Advice Association

Provides advice and information on male and female sexual problems

Web address: www.sda.uk.net

Helpline: 020 7486 7262

Samaritans

Provides support on any issue of concern

Web address: www.samaritans.org

Email address: jo@samaritans.org

National helpline: 08457 909090

Men’s advice line

Provides advice and support for all men experiencing domestic violence

Web address: www.mensadviceline.org.uk

Email address: info@mensadviceline.org.uk

National helpline: 0808 801 0327

National Domestic Violence Helpline
Provides support, help and information to women experiencing domestic violence. It can also be used by family, friends, colleagues and others calling on their behalf.
Web address: www.nationaldomesticviolencehelpline.org.uk
National helpline: 0808 2000 247

London Lesbian and Gay Switchboard
Provides information, support, and referral services
Web address: www.llgs.org.uk
For info on local lines:www.switchboard.org.uk
Email address: admin@llgs.org.uk
Helpline: 0300 330 0630

Relate
Provides counselling and advice on relationship and sexual problems
Web address: www.relate.org.uk
Telephone: 0300 100 1234

Safeline
Provides support for adults who have been sexually abused as children
Website: www.safelinewarwick.co.uk
Email address: office@safelinewarwick.co.uk
Helpline: 0808 800 5005

Rape Crisis
Provides counselling and help for victims of rape and sexual assault
Web address: www.rapecrisis.org.uk
Email address: info@rapecrisis.org.uk
Helpline: 0808 802 9999

British Association for Counselling
Can suggest a local counsellor
Web address: www.bacp.co.uk
Email address: bacp@bacp.co.uk
Telephone: 01455 883 300
