# Supplementary material for: Nonprobability Web Surveys to Measure Sexual Behaviors and Attitudes in the General Population: A Comparison With a Probability Sample Interview Survey
Source: J Med Internet Res. 2014 Dec 8;16(12):e276. doi: 10.2196/jmir.3382 (PMC4275497; doi:10.2196/jmir.3382)
Supplement: Supplementary file 3 [file jmir_v16i12e276_app3.pdf]

| MEN | WS-B1 | WS-B2 | WS-M1 | WS-M2 | Natsal-3 |
|---|---|---|---|---|---|
| Current smoker | 19.7% | 28.8% | 30.2% | 33.9% | 31.5% |
| Drinks more than recommended amount (21+ units in a week) | 8.7% | 8.3% | 8.6% | 8.1% | 7.9% |
| Binge drinks once a week or more (8+ units at one time) | 16.8% | 15.1% | 17.0% | 20.0% | 21.4% |
| 1st sex % <16 | 13.6% | 18.6% | 17.2% | 17.8% | 27.1% |
| 1st sex both equally willing[a] | 89.1% | 86.8% | 88.2% | 88.2% | 91.1% |
| 1st sex contraception used[a] | 90.7% | 86.1% | 86.4% | 87.2% | 82.0% |
| 1st sex was at right time[a] | 63.7% | 62.0% | 60.8% | 64.7% | 74.3% |
| Sex attraction scale – not all opposite sex | 24.4% | 18.2% | 17.1% | 21.1% | 6.7% |
| Sex experience scale – not all opposite sex | 22.2% | 17.0% | 16.3% | 19.8% | 7.1% |
| Had vaginal sex in last month | 53.8% | 56.8% | 62.0% | 60.6% | 70.5% |
| Had het oral sex last year | 63.1% | 66.4% | 71.5% | 70.2% | 79.3% |
| Had het anal sex last year | 13.9% | 18.9% | 20.4% | 17.9% | 17.6% |
| Het sex 5+ occasions last 4 weeks[b] | 42.4% | 52.3% | 51.1% | 48.7% | 46.4% |
| Sex without condom last 4 weeks[c] | 74.4% | 75.3% | 76.9% | 73.6% | 74.9% |
| Ever same-sex sex | 17.1% | 13.2% | 13.3% | 15.5% | 5.0% |
| 1+ same sex partners last 5 yrs | 12.9% | 9.1% | 7.9% | 10.4% | 3.0% |
| STI clinic attendance-last 5 yrs[d] | 16.6% | 14.2% | 14.3% | 10.6% | 18.7% |
| STI clinic attendance-last 1 yr[d] | 5.8% | 5.9% | 5.8% | 4.1% | 6.9% |
| Ever STI diagnosis[d] | 13.4% | 11.2% | 12.3% | 11.1% | 13.3% |
| Any sex problem[e] | 45.3% | 42.7% | 43.7% | 39.7% | 39.5% |
| Ever paid for sex with a woman | 10.8% | 12.4% | 13.2% | 11.3% | 10.4% |
| Heterosexual partners lifetime: | | | | | |
| - None | 17.8% | 12.9% | 11.6% | 10.3% | 6.0% |
| - 1 to 4 | 40.3% | 39.7% | 41.0% | 39.6% | 34.8% |
| - 5 to 9 | 18.0% | 19.4% | 19.8% | 20.5% | 23.6% |
| - 10 + | 24.0% | 28.0% | 27.6% | 29.6% | 35.7% |
| Heterosexual partners last 5 yrs: | | | | | |
| - None | 21.5% | 18.0% | 14.3% | 15.4% | 7.7% |
| - 1 | 42.7% | 42.7% | 46.8% | 45.9% | 46.2% |
| - 2+ | 35.9% | 39.3% | 38.9% | 38.7% | 46.1% |
| Heterosexual partners last 1 year: | | | | | |
| - None | 29.2% | 24.3% | 20.0% | 21.0% | 11.2% |
| - 1 | 54.4% | 61.7% | 61.9% | 64.1% | 68.5% |
| - 2+ | 16.4% | 14.0% | 18.2% | 14.9% | 20.4% |
| 1+ new heterosexual partner last year | 18.9% | 23.9% | 24.7% | 19.3% | 27.4% |
| Ever taken illicit drugs[d] | 53.3% | 45.8% | 44.6% | 49.9% | 52.3% |
| Ever taken cannabis[d] | 49.4% | 41.9% | 40.7% | 45.8% | 49.1% |

Questions in green were asked in CAPI, questions in black were asked in CASI.
a=Mixture of face to face and CASI with 11% answering in CASI
b=Based on those who had opposite-sex sex in the last 4 weeks.
c=Based on those who had opposite- or same-sex sex in the last 4 weeks.
d=The base for these variables is limited to participants who completed the CASI questionnaire.
e=For WS-B2 and WS-M1, these responses are based on half the sample only.

| WOMEN | WS-B1 | WS-B2 | WS-M1 | WS-M2 | Natsal-3 |
|---|---|---|---|---|---|
| Current smoker | 20.0% | 27.5% | 25.5% | 29.2% | 27.1% |
| Drinks more than recommended amount (14+ units in a week) | 13.7% | 12.1% | 9.3% | 16.7% | 11.9% |
| Binge drinks once a week or more (6+units at one time) | 12.9% | 9.8% | 8.6% | 15.7% | 11.8% |
| 1st sex % <16 | 17.5% | 24.3% | 23.8% | 29.4% | 23.5% |
| 1st sex both equally willing[a] | 79.3% | 79.1% | 76.8% | 75.8% | 82.1% |
| 1st sex contraception used[a] | 89.7% | 87.6% | 84.2% | 85.2% | 85.6% |
| 1st sex was at right time[a] | 61.2% | 56.6% | 53.3% | 52.1% | 64.4% |
| Sex attraction scale – not all opposite sex | 34.5% | 28.4% | 29.0% | 34.1% | 16.3% |
| Sex experience scale – not all opposite sex | 27.5% | 24.5% | 24.8% | 29.2% | 15.6% |
| Had vaginal sex in last month | 63.9% | 61.8% | 62.0% | 69.8% | 71.7% |
| Had het oral sex last year | 72.2% | 70.5% | 69.3% | 76.8% | 77.0% |
| Had het anal sex last year | 13.5% | 16.6% | 16.6% | 18.2% | 15.4% |
| Het sex 5+ occasions last 4 weeks[b] | 45.1% | 52.7% | 48.4% | 52.1% | 45.2% |
| Sex without condom last 4 weeks[c] | 80.2% | 78.9% | 81.9% | 82.6% | 80.0% |
| Ever same-sex sex | 14.8% | 13.2% | 13.5% | 14.6% | 8.3% |
| 1+ same sex partners last 5 yrs | 6.6% | 6.4% | 7.2% | 6.2% | 4.9% |
| STI clinic attendance-last 5 yrs[d] | 19.3% | 18.4% | 18.7% | 17.5% | 20.3% |
| STI clinic attendance-last 1 yr[d] | 6.8% | 6.0% | 6.4% | 6.9% | 8.5% |
| Ever STI diagnosis[d] | 16.4% | 17.2% | 18.3% | 18.1% | 18.8% |
| Any sex problem[e] | 57.5% | 50.6% | 57.2% | 54.5% | 48.3% |
| Ever abortion[4] | 13.8% | 14.7% | 15.2% | 18.6% | 14.8% |
| Heterosexual partners lifetime: | | | | | |
| - None | 8.0% | 5.9% | 8.0% | 5.9% | 4.5% |
| - 1 to 4 | 41.4% | 41.9% | 43.5% | 37.3% | 43.0% |
| - 5 to 9 | 20.6% | 24.7% | 22.5% | 25.5% | 26.0% |
| - 10 + | 30.0% | 27.6% | 26.0% | 31.4% | 26.6% |
| Heterosexual partners Last 5 years: - None | 12.3% | 9.9% | 13.1% | 8.4% | 6.4% |
| - 1 | 53.0% | 53.6% | 54.2% | 55.6% | 55.4% |
| - 2+ | 34.7% | 36.5% | 32.7% | 35.9% | 38.3% |
| Heterosexual partners Last 1 year: | | | | | |
| - None | 18.2% | 17.7% | 19.6% | 12.7% | 10.7% |
| - 1 | 70.2% | 72.3% | 70.9% | 75.2% | 74.5% |
| - 2+ | 11.7% | 10.0% | 9.5% | 12.1% | 14.7% |
| 1+ new heterosexual partner last year | 14.8% | 17.4% | 15.3% | 17.7% | 21.8% |
| Ever taken illicit drugs[d] | 47.3% | 40.6% | 41.3% | 49.4% | 38.5% |
| Ever taken cannabis[d] | 44.5% | 36.8% | 37.9% | 46.0% | 35.5% |

Questions in green were asked in CAPI, questions in black were asked in CASI.
a=Mixture of face to face and CASI with 11% answering in CASI
b=Based on those who had opposite-sex sex in the last 4 weeks.
c=Based on those who had opposite- or same-sex sex in the last 4 weeks.
d=The base for these variables is limited to participants who completed the CASI questionnaire.
e=For WS-B2 and WS-M1, these responses are based on half the sample only.
