# Supplementary material for: Nonprobability Web Surveys to Measure Sexual Behaviors and Attitudes in the General Population: A Comparison With a Probability Sample Interview Survey
Source: J Med Internet Res. 2014 Dec 8;16(12):e276. doi: 10.2196/jmir.3382 (PMC4275497; doi:10.2196/jmir.3382)
Supplement: Supplementary file 4 [file jmir_v16i12e276_app4.pdf]

	WS-M1<br>Achieved/target (ratio)	WS-M2<br>Achieved/target (ratio)
**Age within sex**		
**Men**		
18-24	268/267 (1.00)	228/267 (0.85)
25-34	352/360 (0.98)	420/360 (1.17)
35-44	389/382 (1.02)	382/382 (1.0)
**Women**		
18-24	252/252 (1.0)	252/252 (1.0)
25-34	350/350 (1.0)	350/350 (1.0)
35-44	389/389 (1.0)	389/389 (1.0)
**Partnership status within sex**		
**Men**		
Married/living as married	555/555 (1.0)	562/555 (1.01)
All others	454/454 (1.0)	468/454 (1.03)
**Women**		
Married/living as married	565/565 (1.0)	565/565 (1.0)
All others	426/426 (1.0)	426/426 (1.0)
**Region**		
London	318/320 (0.99)	n.a.
All others	1682/1680 (1.00)	n.a.
**Age left full-time education**		
16 or under	550/640 (0.86)	577/640 (0.90)
17-19	553/520 (1.06)	547/520 (1.05)
20+	642/600 (1.07)	641/600 (1.07)
Still in full-time education	255/240 (1.06)	256/240 (1.07)
**Any <18s in household**		
None	994/1000 (0.99)	n.a.
1+	1006/1000 (1.01)	n.a.
**Persons living in household**		
1	n.a.	208/200 (1.04)
2 - 3	n.a.	989/940 (1.05)
4 or more	n.a.	824/860 (0.96)
**Frequency of drinking alcohol**		
3+ times per week	n.a.	406/420 (0.97)
1-2 per week	n.a.	923/860 (1.07)
Less often	n.a.	692/720 (0.96)
**Attitude to same-gender sex**		
Always/mostly wrong	n.a.	469/540 (0.87)
Sometimes/rarely/never wrong	n.a.	1444/1380 (1.05)
Depends/don’t know	n.a.	108/80 (1.35)

n.a. = not applicable
