# Supplementary material for: Nonprobability Web Surveys to Measure Sexual Behaviors and Attitudes in the General Population: A Comparison With a Probability Sample Interview Survey
Source: J Med Internet Res. 2014 Dec 8;16(12):e276. doi: 10.2196/jmir.3382 (PMC4275497; doi:10.2196/jmir.3382)
Supplement: Supplementary file 5 [file jmir_v16i12e276_app5.pdf]

MEN	WS-B1	WS-B2	WS-M1	WS-M2	Natsal-3	General population
**Age group[a]**						
18-24	26.0%	26.0%	26.0%	26.0%	26.0%	26.0%
25-34	36.3%	36.3%	36.3%	36.3%	36.3%	36.3%
35-44	37.7%	37.7%	37.7%	37.7%	37.7%	37.7%
**Marital status[a,d]**						
Married, living with spouse	28.3%	35.1%	43.7%	31.1%	37.7%	31.3%
Other	71.7%	64.9%	56.3%	68.9%	62.3%	68.7%
**Age completed full-time education[b]**						
Under 16	4.0%	5.0%	6.1%	4.4%	6.7%	5.0%
16-19	31.2%	43.7%	48.9%	49.2%	51.0%	56.2%
20+	49.8%	38.8%	32.1%	34.7%	29.8%	27.8%
Still in full-time education	15.0%	12.5%	12.9%	11.6%	12.5%	10.8%
**Household size[b]**						
One	16.6%	14.3%	13.5%	11.7%	12.6%	24.0%
Two	31.7%	27.9%	23.6%	29.7%	23.5%	22.1%
Three +	51.7%	57.8%	62.9%	58.6%	64.0%	53.9%
**Current economic activity[a,d,e]**						
Still in full-time education	13.9%	11.1%	13.2%	9.2%	9.9%	7.1%
In employment	75.8%	70.4%	68.5%	74.9%	77.1%	78.2%
Looking after family/home	1.6%	4.3%	3.9%	4.3%	1.2%	0.9%
Other activity	8.8%	14.2%	14.4%	11.6%	11.8%	13.8%
**Sexual identity[b]**						
Heterosexual	84.8%	89.4%	90.3%	88.8%	96.9%	96.7%
Gay	8.8%	5.8%	2.8%	7.9%	1.8%	2.4%
Bisexual/other	6.4%	4.8%	6.9%	3.3%	1.2%	0.9%
**Driving licence valid in UK[c]**						
Yes	78.6%	70.1%	70.4%	73.3%	73.2%	75.7%
No	21.4%	29.9%	29.6%	26.7%	26.8%	24.3%
**Tenure[b]**						
Own/mortgage	58.5%	49.7%	55.2%	49.2%	51.9%	56.7%
Other	41.5%	50.3%	44.8%	50.8%	48.1%	43.3%
**General health[a,d]**						
Very good/good	78.9%	80.2%	78.7%	71.4%	88.7%	91.2%
Fair/bad/very bad	21.1%	19.8%	21.3%	28.6%	11.3%	8.8%
**Ethnicity[a,d]**						
White	91.3%	84.8%	84.5%	92.8%	81.8%	81.9%
Mixed	1.9%	2.0%	2.2%	0.9%	2.4%	2.2%
Asian	4.4%	9.5%	9.3%	4.0%	9.6%	10.4%
Black	1.0%	1.8%	2.9%	1.3%	4.3%	3.8%
Chinese/other	1.4%	1.8%	1.1%	0.9%	1.8%	1.6%

Sources of general population data:
a=Census 2011; b=Integrated Household Survey 2011; c=National Travel Survey 2010. d=Data for England & Wales only.
e=For current economic activity, some of the differences in the estimates between the web surveys, Natsal-3 and census may be due to small variations in the way the questions were asked and coded.

WOMEN	WS-B1	WS-B2	WS-M1	WS-M2	Natsal-3	General population
**Age group[a]**						
18-24	25.3%	25.3%	25.3%	25.3%	25.3%	25.3%
25-34	36.4%	36.4%	36.4%	36.4%	36.4%	36.4%
35-44	38.4%	38.4%	38.4%	38.4%	38.4%	38.4%
**Marital status[a,d]**						
Married, living with spouse	34.6%	39.9%	43.3%	39.9%	40.0%	35.9%
Other	65.4%	60.1%	56.7%	60.1%	60.0%	64.1%
**Age completed full-time education[b]**						
Under 16	3.8%	3.0%	6.8%	5.7%	6.1%	5.2%
16-19	31.5%	48.1%	48.2%	52.3%	51.0%	55.3%
20+	48.6%	34.9%	32.6%	26.5%	30.9%	29.4%
Still in full-time education	16.1%	14.0%	12.5%	15.6%	12.0%	9.8%
**Household size[b]**						
One	12.5%	11.2%	12.7%	5.1%	8.1%	13.3%
Two	32.2%	28.6%	27.2%	23.9%	23.7%	26.9%
Three +	55.3%	60.2%	60.0%	71.0%	68.2%	59.8%
**Current economic activity[a,d,e]**						
Still in full-time education	13.5%	12.2%	9.0%	10.0%	8.8%	7.0%
In employment	67.9%	58.2%	56.9%	60.1%	65.1%	70.3%
Looking after family/home	11.9%	19.0%	21.0%	18.9%	17.7%	10.9%
Other activity	6.7%	10.6%	13.1%	11.1%	8.4%	11.7%
**Sexual identity[b]**						
Heterosexual	90.8%	92.3%	92.5%	92.6%	96.1%	97.9%
Gay	2.2%	1.2%	2.3%	1.3%	1.3%	1.1%
Bisexual/other	7.0%	6.5%	5.2%	6.1%	2.6%	1.0%
**Driving licence valid in UK[c]**						
Yes	76.1%	66.2%	62.7%	68.2%	70.5	68.8%
No	23.9%	33.8%	37.3%	31.8%	29.5	31.2%
**Tenure[b]**						
Own/mortgage	54.7%	47.8%	49.0%	47.2%	50.8%	55.5%
Other	45.3%	52.2%	51.0%	52.8%	49.2%	44.5%
**General health[a,d]**						
Very good/good	78.3%	78.9%	75.9%	74.0%	87.9%	90.2%
Fair/bad/very bad	21.7%	21.1%	24.1%	26.0%	12.1%	9.8%
**Ethnicity[a,d]**						
White	94.3%	87.4%	86.6%	90.3%	82.7%	82.2%
Mixed	2.0%	2.5%	1.7%	2.4%	2.7%	2.3%
Asian	2.1%	6.5%	7.0%	2.6%	8.2%	10.1%
Black	0.6%	2.6%	3.0%	2.8%	4.6%	4.2%
Chinese/other	1.0%	1.0%	1.6%	1.9%	1.8%	1.2%

Sources of general population data:
a=Census 2011; b=Integrated Household Survey 2011; c=National Travel Survey 2010. d=Data for England & Wales only.
e=For current economic activity, some of the differences in the estimates between the web surveys, Natsal-3 and census may be due to small variations in the way the questions were asked and coded.
