# Supplementary material for: Nonprobability Web Surveys to Measure Sexual Behaviors and Attitudes in the General Population: A Comparison With a Probability Sample Interview Survey
Source: J Med Internet Res. 2014 Dec 8;16(12):e276. doi: 10.2196/jmir.3382 (PMC4275497; doi:10.2196/jmir.3382)
Supplement: Supplementary file 6 [file jmir_v16i12e276_app6.pdf]

MEN	WS-B1	WS-B2	WS-M1	WS-M2	Natsal-3
Current economic activity: in employment[a,d,e]	0.88 [0.74,1.03]	0.66 [0.57,0.76]	0.61 [0.53,0.69]	0.83 [0.72,0.97]	0.94 [0.85,1.03]
Sexual identity: not heterosexual[b]	5.23 [4.37,6.19]	3.45 [2.81,4.20]	3.13 [2.52,3.83]	3.66 [3.00,4.43]	0.92 [0.72,1.16]
Has driving licence valid in UK[c]	1.17 [0.98,1.39]	0.79 [0.67,0.92]	0.77 [0.65,0.90]	0.88 [0.75,1.04]	1.14 [0.94,1.39]
Tenure: own/ mortgage[b]	1.08 [0.95,1.23]	0.75 [0.67,0.85]	0.94 [0.83,1.07]	0.74 [0.65,0.84]	0.82 [0.76,0.89]
General health: very good/ good[a,d]	0.36 [0.31,0.42]	0.39 [0.33,0.46]	0.36 [0.31,0.42]	0.24 [0.21,0.28]	0.76 [0.67,0.86]
Ethnicity: non-white[a,d]	0.43 [0.34,0.54]	0.81 [0.67,0.97]	0.83 [0.70,0.99]	0.35 [0.28,0.45]	1.00 [0.89,1.13]
**WOMEN**					
Current economic activity: in employment[a,d,e]	0.89 [0.78,1.02]	0.59 [0.51,0.67]	0.56 [0.49,0.63]	0.63 [0.56,0.72]	0.79 [0.73,0.84]
Sexual identity: not heterosexual[b]	4.78 [3.84,5.84]	3.94 [3.09,4.93]	3.83 [3.00,4.81]	3.77 [2.94,4.75]	1.91 [1.61,2.74]
Has driving licence valid in UK[c]	1.44 [1.23,1.69]	0.88 [0.76,1.03]	0.76 [0.66,0.88]	0.97 [0.83,1.13]	0.92 [0.79,1.08]
Tenure: own/ mortgage[b]	0.97 [0.86,1.10]	0.74 [0.65,0.84]	0.77 [0.68,0.88]	0.72 [0.63,0.82]	0.83 [0.78,0.89]
General health: very good/ good[a,d]	0.39 [0.34,0.46]	0.41 [0.35,0.48]	0.34 [0.30,0.40]	0.31 [0.27,0.36]	0.79 [0.72,0.88]
Ethnicity: non-white[a,d]	0.28 [0.21,0.37]	0.67 [0.55,0.81]	0.71 [0.59,0.86]	0.50 [0.40,0.62]	0.96 [0.87,1.07]

Sources of general population data:

a=Census 2011; b=Integrated Household Survey 2011; c=National Travel Survey 2010. d=Data for England & Wales only.

e=For current economic activity, some of the differences in the estimates between the web surveys, Natsal-3 and census may be due to small variations in the way the questions were asked and coded.
