# Supplementary material for: Nonprobability Web Surveys to Measure Sexual Behaviors and Attitudes in the General Population: A Comparison With a Probability Sample Interview Survey
Source: J Med Internet Res. 2014 Dec 8;16(12):e276. doi: 10.2196/jmir.3382 (PMC4275497; doi:10.2196/jmir.3382)
Supplement: Supplementary file 7 [file jmir_v16i12e276_app7.pdf]

MEN	WS-B1	WS-B2	WS-M1	WS-M2	Natsal-3
**One night stands**					
Always wrong	12.0%	17.8%	16.2%	12.4%	21.1%
Mostly wrong	14.9%	14.7%	16.7%	14.5%	14.6%
Sometimes wrong	26.2%	25.5%	21.0%	30.0%	30.2%
Rarely wrong	11.5%	8.7%	9.4%	12.4%	10.4%
Not wrong	19.6%	17.2%	19.9%	17.6%	19.8%
Depends/DK	15.8%	16.1%	16.9%	13.1%	4.0%
**Adultery**					
Always wrong	38.0%	45.2%	46.0%	38.5%	61.7%
Mostly wrong	38.0%	29.4%	27.8%	36.5%	24.9%
Sometimes wrong	10.7%	12.3%	11.8%	13.5%	9.7%
Rarely wrong	1.2%	1.0%	2.1%	1.9%	1.1%
Not wrong	1.9%	3.0%	3.2%	1.5%	1.5%
Depends/DK	10.3%	9.1%	9.1%	8.2%	1.2%
**Sex between men**					
Always wrong	13.4%	23.2%	23.5%	14.9%	26.6%
Mostly wrong	4.6%	7.8%	7.0%	9.8%	8.3%
Sometimes wrong	5.5%	6.0%	7.2%	9.7%	7.4%
Rarely wrong	8.8%	5.7%	5.5%	8.0%	7.2%
Not wrong	54.4%	39.3%	40.0%	50.5%	46.4%
Depends/DK	13.3%	18.0%	16.9%	7.2%	4.0%
**Sex between women**					
Always wrong	7.5%	14.1%	14.1%	8.1%	17.5%
Mostly wrong	4.7%	7.1%	6.9%	6.9%	8.8%
Sometimes wrong	7.8%	7.7%	8.3%	9.9%	10.1%
Rarely wrong	8.9%	8.8%	7.4%	11.8%	9.1%
Not wrong	59.0%	45.7%	48.2%	54.6%	50.5%
Depends/DK	12.2%	16.7%	15.1%	8.8%	4.1%
**Talk to parents about sex**					
Easy	8.9%	13.4%	16.1%	15.0%	18.4%
Difficult	12.5%	13.4%	13.8%	11.7%	9.1%
Didn't	69.8%	65.7%	59.5%	64.2%	69.9%
Depends on topic	8.8%	7.5%	10.7%	9.1%	2.7%
**Satisfied with sex life**[a]					
Agree strongly	13.3%	21.4%	19.8%	17.3%	25.2%
Agree	29.3%	31.8%	36.9%	32.6%	40.5%
Neither	23.0%	20.7%	15.8%	20.2%	17.8%
Disagree	22.7%	16.7%	17.0%	18.2%	13.5%
Disagree strongly	11.2%	9.5%	9.8%	11.7%	3.0%
**Distressed about sex life**[a]					
Agree strongly	2.4%	4.5%	3.7%	2.8%	1.7%
Agree	12.3%	14.6%	16.3%	16.2%	8.1%
Neither	26.4%	22.4%	21.7%	23.4%	18.3%
Disagree	36.8%	31.1%	28.8%	28.5%	37.9%
Disagree strongly	22.1%	27.5%	29.5%	29.1%	34.0%
**Avoided sex because of problems**[a]					
Agree strongly	1.6%	2.4%	3.2%	3.2%	1.4%
Agree	13.3%	11.7%	11.8%	9.2%	7.7%
Neither	16.7%	15.6%	13.6%	15.9%	10.4%
Disagree	32.2%	27.4%	27.5%	24.6%	33.0%
Disagree strongly	36.3%	43.0%	43.9%	47.2%	47.4%

Questions in green were asked in CAPI, questions in black were asked in CASI.
a=The base for these questions is limited to participants who answered the CASI questionnaire.

WOMEN	WS-B1	WS-B2	WS-M1	WS-M2	Natsal-3
**One night stands**					
Always wrong	15.3%	19.9%	22.7%	18.4%	29.0%
Mostly wrong	18.8%	20.1%	18.6%	19.2%	18.4%
Sometimes wrong	27.0%	26.1%	26.7%	28.7%	28.2%
Rarely wrong	8.4%	5.6%	5.9%	8.2%	7.2%
Not wrong	14.7%	13.2%	11.3%	11.8%	12.9%
Depends/DK	15.8%	15.1%	14.9%	13.9%	4.3%
**Adultery**					
Always wrong	54.0%	58.7%	60.5%	56.8%	68.8%
Mostly wrong	34.0%	26.3%	23.6%	31.4%	21.6%
Sometimes wrong	6.4%	7.7%	6.4%	6.1%	7.3%
Rarely wrong	0.7%	0.5%	0.9%	0.7%	0.5%
Not wrong	0.6%	1.2%	2.0%	0.7%	0.7%
Depends/DK	4.4%	5.6%	6.6%	4.3%	1.1%
**Sex between men**					
Always wrong	7.0%	11.7%	13.7%	13.9%	13.5%
Mostly wrong	2.1%	5.2%	3.4%	7.8%	4.6%
Sometimes wrong	4.5%	5.1%	5.0%	6.2%	6.1%
Rarely wrong	7.9%	7.1%	7.8%	10.2%	8.2%
Not wrong	64.3%	56.0%	55.6%	58.4%	63.9%
Depends/DK	14.3%	15.0%	14.5%	3.5%	3.7%
**Sex between women**					
Always wrong	6.3%	11.9%	13.4%	15.5%	13.5%
Mostly wrong	1.9%	4.4%	3.8%	6.4%	4.5%
Sometimes wrong	4.8%	5.6%	4.6%	6.2%	6.2%
Rarely wrong	8.2%	7.5%	7.7%	9.7%	8.4%
Not wrong	65.0%	56.3%	56.1%	58.1%	63.8%
Depends/DK	13.8%	14.3%	14.4%	4.3%	3.7%
**Talk to parents about sex**					
Easy	15.9%	14.9%	15.0%	15.4%	25.2%
Difficult	12.1%	14.4%	14.2%	12.7%	12.3%
Didn't	58.1%	58.3%	58.3%	59.9%	59.0%
Depends on topic	14.0%	12.4%	12.6%	11.9%	3.5%
**Satisfied with sex life**[a]					
Agree strongly	15.6%	21.6%	18.7%	17.5%	27.3%
Agree	35.3%	33.6%	34.0%	33.7%	39.8%
Neither	20.3%	20.1%	21.0%	19.4%	19.9%
Disagree	18.9%	15.4%	17.0%	19.3%	10.2%
Disagree strongly	9.4%	9.3%	8.9%	10.1%	2.9%
**Distressed about sex life**[a]					
Agree strongly	3.6%	3.5%	3.6%	4.5%	1.7%
Agree	16.7%	14.9%	16.0%	17.1%	9.4%
Neither	24.0%	22.7%	23.3%	21.2%	20.2%
Disagree	33.5%	30.2%	30.3%	30.3%	35.9%
Disagree strongly	22.2%	28.7%	26.8%	26.9%	32.7%
**Avoided sex because of problems**[a]					
Agree strongly	4.3%	2.9%	3.6%	4.2%	2.0%
Agree	15.0%	14.7%	14.8%	13.0%	8.9%
Neither	14.8%	13.1%	17.4%	13.8%	11.7%
Disagree	30.9%	29.3%	26.6%	27.4%	35.9%
Disagree strongly	35.1%	40.1%	37.7%	41.6%	41.5%

Questions in green were asked in CAPI, questions in black were asked in CASI.
a=The base for these questions is limited to participants who answered the CASI questionnaire.
