# Supplementary material for: Nonprobability Web Surveys to Measure Sexual Behaviors and Attitudes in the General Population: A Comparison With a Probability Sample Interview Survey
Source: J Med Internet Res. 2014 Dec 8;16(12):e276. doi: 10.2196/jmir.3382 (PMC4275497; doi:10.2196/jmir.3382)
Supplement: Supplementary file 8 [file jmir_v16i12e276_app8.pdf]

MEN	WS-B1	WS-B2	WS-M1	WS-M2
Current smoker	0.53 [0.45,0.64]	0.88 [0.75,1.03]	0.94 [0.80,1.10]	1.12 [0.96,1.31]
Drinks more than recommended amount (21+ units in a week)	1.11 [0.86,1.43]	1.04 [0.80,1.36]	1.10 [0.84,1.42]	1.02 [0.78,1.33]
Binge drinks once a week or more (8+ units)	0.75 [0.62,0.90]	0.65 [0.54,0.79]	0.75 [0.62,0.91]	0.92 [0.77,1.10]
1st sex % <16	0.40 [0.32,0.49]	0.55 [0.45,0.66]	0.52 [0.43,0.64]	0.55 [0.45,0.66]
1st sex both equally willing[a]	0.80 [0.61,1.05]	0.65 [0.51,0.83]	0.73 [0.57.0.94]	0.74 [0.57,0.94]
1st sex contraception used[a]	2.14 [1.66,2.77]	0.73 [0.59,0.92]	0.72 [0.57,0.90]	0.67 [0.53,0.84]
1st sex was at right time[a]	0.61 [0.51,0.72]	0.56 [0.48,0.67]	0.54 [0.46,0.64]	0.63 [0.54,0.75]
Sex attraction scale – not all opposite sex	4.54 [3.70,5.57]	3.11 [2.51,3.86]	2.88 [2.31,3.58]	3.76 [3.06,4.62]
Sex experience scale – not all opposite sex	3.71 [2.99,4.62]	2.68 [2.13,3.37]	2.52 [2.00,3.17]	3.23 [2.59,4.02]
Had vaginal sex last month	0.49 [0.42,0.57]	0.55 [0.47,0.64]	0.68 [0.59,0.80]	0.65 [0.55,0.75]
Had het oral sex last year	0.45 [0.38,0.52]	0.52 [0.44,0.61]	0.66 [0.56,0.78]	0,.62 [0.52,0.73]
Had het anal sex last year	0.75 [0.61,0.93]	1.09 [0.91,1.32]	1.20 [1.00,1.44]	1.02 [0.85,1.23]
Het sex 5+ occasions last 4 weeks[b]	1.00 [0.84,1.18]	1.35 [1.13,1.61]	1.14 [0.96,1.36]	1.32 [1.12,1.56]
Sex without condom last 4 weeks[c]	1.02 [0.82,1.28]	0.98 [0.79,1.22]	0.90 [0.72,1.11]	1.07 [0.87,1.32]
Ever same-sex sex	3.89 [3.04,4.98]	2.87 [2.22,3.72]	2.89 [2.23,3.74]	3.45 [2.69,4.43]
1+ same sex partners last 5 yrs	4.85 [3.63,6.47]	3.28 [2.41,4.46]	2.81 [2.04,3.86]	3.78 [2.80,5.09]
STI clinic attendance-last 5 years[d]	0.88 [0.72,1.07]	0.72 [0.58,0.89]	0.72 [0.59,0.89]	0.51 [0.41,0.65]
STI clinic attendance-last 1 year[d]	0.85 [0.63,1.15]	0.85 [0.62,1.15]	0.83 [0.61,1.13]	0.57 [0.40,0.82]
Ever STI diagnosis[d]	0.98 [0.78,1.22]	0.80 [0.63,1.01]	0.80 [0.64,1.00]	0.79 [0.62,1.00]
Any sex problem[e]	1.27 [1.08,1.50]	1.14 [0.92,1.41]	1.19 [0.96,1.48]	1.01 [0.86,1.19]
Ever paid for sex with a woman	0.99 [0.78,1.26]	1.17 [0.92,1.48]	1.24 [0.98,1.57]	1.07 [0.83,1.36]
Heterosexual partners lifetime	0.87 [0.76,0.99]	0.99 [0.87,1.13]	0.85 [0.74,0.98]	1.18 [1.03,1.35]
Heterosexual partners last 5 yrs	0.69 [0.60,0.80]	0.86 [0.75,1.00]	0.69 [0.59,0.80]	0.88 [0.76,1.01]
Heterosexual partners last 1 year	0.63 [0.54,0.74]	0.59 [0.50,0.70]	0.53 [0.45,0.63]	0.81 [0.70,0.95]
1+ new heterosexual partner last year	0.63 [0.52,0.76]	0.83 [0.70,0.99]	0.89 [0.75,1.05]	0.65 [0.54,0.78]
Ever taken illicit drugs[d]	1.04 [0.89,1.21]	0.77 [0.66,0.90]	0.73 [0.63,0.86]	0.91 [0.78,1.05]
Ever taken cannabis[d]	1.01 [0.87,1.18]	0.75 [0.64,0.87]	0.71 [0.61,0.83]	0.88 [0.75,1.02]
One night stands always/mostly wrong	0.67 [0.57,0.78]	0.87 [0.74,1.01]	0.89 [0.76,1.03]	0.67 [0.57,0.78]
Adultery always/mostly wrong	0.49 [0.41,0.59]	0.46 [0.38,0.55]	0.44 [0.37,0.53]	0.47 [0.39,0.56]
Sex between men always/mostly wrong	0.41 [0.34,0.49]	0.84 [0.71,0.98]	0.82 [0.70,0.96]	0.61 [0.52,0.72]
Sex between women always/ mostly wrong	0.39 [0.31,0.48]	0.75 [0.63,0.90]	0.75 [0.62,0.90]	0.49 [0.40,0.60]
Easy to talk to parents about sex	0.43 [0.34,0.55]	0.69[0.56,0.85]	0.85 [0.70,1.04]	0.78 [0.64,0.96]
Satisfied with sex life agree/strongly[d]	0.39 [0.34,0.46]	0.59 [0.51,0.69]	0.70 [0.60,0.81]	0.52 [0.44,0.60]
Distressed about sex life agree/strongly[d]	1.60 [1.28,1.99]	2.18 [1.78,2.67]	2.24 [1.83,2.75]	2.12 [1.73,2.60]
Avoided sex because of problems agree/strongly[d]	1.74 [1.39,2.18]	1.63 [1.29,2.05]	1.68 [1.33,2.12]	1.37 [1.08,1.74]

Questions in green were asked in CAPI, questions in black were asked in CASI.
a=Mixture of face to face and CASI with 11% answering in CASI
b=Based on those who had opposite-sex sex in the last 4 weeks.
c=Based on those who had opposite- or same-sex sex in the last 4 weeks.
d=The base for these variables is limited to participants who completed the CASI questionnaire.
e=For WS-B2 and WS-M1, these responses are based on half the sample only.

WOMEN	WS-B1	WS-B2	WS-M1	WS-M2
Current smoker	0.67 [0.57,0.79]	1.02 [0.88,1.19]	0.92 [0.79,1.08]	1.11 [0.95,1.29]
Drinks more than recommended amount (14+ units in a week)	1.18 [0.96,1.44]	1.02 [0.83,1.27]	0.76 [0.60,0.97]	1.49 [1.22,1.81]
Binge drinks once a week or more (6+ units)	1.11 [0.91,1.36]	0.81 [0.65,1.02]	0.70 [0.55,0.88]	1.40 [1.15,1.70]
1st sex % <16	0.67 [0.56,0.80]	1.03 [0.87,1.22]	0.98 [0.83,1.16]	1.30 [1.11,1.53]
1st sex both equally willing[a]	0.83 [0.70,1.00]	0.82 [0.68,0.99]	0.72 [0.60,0.86]	0.68 [0.57,0.81]
1st sex contraception used[a]	1.46 [1.16,1.84]	0.84 [0.67,1.05]	1.11 [0.91,1.37]	1.03 [0.84,1.27]
1st sex was at right time[a]	0.87 [0.75,1.01]	0.72 [0.61,0.84]	0.63 [0.54,0.73]	0.60 [0.52,0.70]
Sex attraction scale – not all opposite sex	2.70 [2.32,3.15]	2.04 [1.73,2.4]	2.09 [1.77,2.46]	2.65 [2.27,3.11]
Sex experience scale – not all opposite sex	2.06 [1.75,2.42]	1.76 [1.48,2.08]	1.79 [1.51,2.12]	2.23 [1.89,2.63]
Had vaginal sex last month	0.70 [0.61,0.08]	0.64 [0.55,0.74]	0.65 [0.56,0.75]	0.91 [0.78,1.06]
Had het oral sex last year	0.78 [0.67,0.91]	0.72 [0.61,0.84]	0.68 [0.58,0.79]	0.99 [0.84,1.17]
Had het anal sex last year	0.86 [0.70,1.04]	1.09 [0.91,1.32]	1/10 [0.91,1.32]	1.23 [1.02,1.48]
Het sex 5+ occasions last 4 weeks[b]	0.85 [0.70,1.03]	1.27 [1.05,1.53]	1.21 [1.01,1.45]	1.10 [0.92,1.32]
Sex without condom last 4 weeks[c]	0.98 [0.80,1.22]	1.07 [0.85,1.33]	0.88 [0.70,1.11]	0.84 [0.68,1.05]
Ever same-sex sex	1.91 [1.56,2.34]	1.68 [1.35,2.09]	1.72 [1.39,2.13]	1.89 [1.53,2.33]
1+ same sex partners last 5 yrs	1.37 [1.03,1.81]	1.33 [1.00,1.79]	1.50 [1.13,1.98]	1.27 [0.95,1.71]
STI clinic attendance-last 5 years[d]	0.96 [0.81,1.14]	0.89 [0.74,1.07]	0.91 [0.76,1.09]	0.83 [0.69,1.01]
STI clinic attendance-last 1 year[d]	0.80 [0.61,1.05]	0.69 [0.51,0.92]	0.74 [0.55,0.98]	0.80 [0.60,1.06]
Ever STI diagnosis[d]	0.85 [0.70,1.02]	0.89 [0.74,1.08]	0.89 [0.74,1.07]	0.95 [0.79,1.15]
Any sex problem[e]	1.45 [1.25,1.68]	1.10 [0.90,1.34]	1.43 [1.16,1.77]	1.31 [1.09,1.58]
Ever abortion[d]	0.92 [0.75,1.12]	0.99 [0.81,1.21]	1.03 [0.84,1.26]	1.28 [1.11,1.49]
Heterosexual partners lifetime	0.44 [0.38,0.51]	0.61 [0.53,0.71]	0.62 [0.54,0.72]	0.70 [0.60,0.80]
Heterosexual partners last 5 yrs	0.49 [0.42,0.58]	0.64 [0.55,0.74]	0.68 [0.59,0.79]	0.66 [0.57,0.77]
Heterosexual partners last 1 year	0.45 [0.37,0.53]	0.48 [0.41,0.56]	0.67 [0.57,0.78]	0.56 [0.48,0.66]
1+ new heterosexual partner last year	0.64 [0.53,0.77]	0.75 [0.63,0.91]	0.66 [0.55,0.80]	0.79 [0.66,0.94]
Ever taken illicit drugs[d]	1.43 [1.25,1.65]	1.10 [0.95,1.27]	1.13 [0.98,1.30]	1.56 [1.36,1.80]
Ever taken cannabis[d]	1.46 [1.27,1.68]	1.06 [0.91,1.23]	1.11 [0.96,1.29]	1.55 [1.34,1.79]
One night stands always/mostly wrong	0.58 [0.50,0.66]	0.60 [0.49,0.74]	0.78 [0.68,0.90]	0.67 [0.58,0.77]
Adultery always/mostly wrong	0.78 [0.63,0.96]	0.74 [0.64,0.86]	0.56 [0.46,0.69]	0.80 [0.64,1.00]
Sex between men always/mostly wrong	0.45 [0.36,0.57]	0.92 [0.76,1.11]	0.93 [0.77,1.13]	1.26 [1.06,1.50]
Sex between women always/ mostly wrong	0.41 [0.32,0.52]	0.89 [0.73,1.08]	0.95 [0.79,1.15]	1.28 [1.07,1.52]
Easy to talk to parents about sex	0.56 [0.47,0.67]	0.52 [0.43,0.63]	0.52 [0.43,0.63]	0.54 [0.45,0.65]
Satisfied with sex life agree/ strongly[d]	0.51 [0.44,0.58]	0.61 [0.52,0.70]	0.56 [0.48,0.65]	0.52 [0.45,0.60]
Distressed about sex life agree/strongly[d]	2.02 [1.68,2.42]	1.79 [1.48,2.18]	1.91 [1.58,2.32]	2.20 [1.83,2.65]
Avoided sex because of problems agree/strongly[d]	1.94[1.61,2.34]	1.74 [1.42,2.12]	1.82 [1.50,2.22]	1.70 [1.39,2.07]

Questions in green were asked in CAPI, questions in black were asked in CASI.
a=Mixture of face to face and CASI with 11% answering in CASI
b=Based on those who had opposite-sex sex in the last 4 weeks.
c=Based on those who had opposite- or same-sex sex in the last 4 weeks.
d=The base for these variables is limited to participants who completed the CASI questionnaire.
e=For WS-B2 and WS-M1, these responses are based on half the sample only.
